# CCL3/Macrophage Inflammatory Protein-1α Is Dually Involved in Parasite Persistence and Induction of a TNF- and IFNγ-Enriched Inflammatory Milieu in *Trypanosoma cruzi*-Induced Chronic Cardiomyopathy

**DOI:** 10.3389/fimmu.2020.00306

**Published:** 2020-03-03

**Authors:** Daniel Gibaldi, Glaucia Vilar-Pereira, Isabela Resende Pereira, Andrea Alice Silva, Leda Castaño Barrios, Isalira Peroba Ramos, Hílton Antônio Mata dos Santos, Ricardo Gazzinelli, Joseli Lannes-Vieira

**Affiliations:** ^1^Laboratório de Biologia das Interações, Instituto Oswaldo Cruz (Fiocruz), Rio de Janeiro, Brazil; ^2^Laboratório Multiusuário de Apoio à Pesquisa em Nefrologia e Ciências Médicas, Departamento de Patologia, Faculdade de Medicina, Universidade Federal Fluminense, Rio de Janeiro, Brazil; ^3^Laboratório de Cardiologia Celular e Molecular, Instituto de Biofísica Carlos Chagas Filho, Universidade Federal do Rio de Janeiro, Rio de Janeiro, Brazil; ^4^Centro Nacional de Biologia Estrutural e Bioimagem, Centro de Ciências da Saúde (CCS), Universidade Federal do Rio de Janeiro, Rio de Janeiro, Brazil; ^5^Faculdade de Farmácia, Universidade Federal do Rio de Janeiro, Rio de Janeiro, Brazil; ^6^Departamento de Bioquímica e Imunologia, Universidade Federal de Minas Gerais, Belo Horizonte, Brazil

**Keywords:** Chagas disease, heart disease, cell migration, CCL3, IFNγ, TNF, Met-RANTES, QTc prolongation

## Abstract

CCL3, a member of the CC-chemokine family, has been associated with macrophage recruitment to heart tissue and parasite control in the acute infection of mouse with *Trypanosoma cruzi*, the causative agent of Chagas disease. Here, we approached the participation of CCL3 in chronic chagasic cardiomyopathy (CCC), the main clinical form of Chagas disease. We induced CCC in C57BL/6 (*ccl*3^+/+^) and CCL3-deficient (*ccl*3^−/−^) mice by infection with the Colombian Type I strain. In *ccl*3^+/+^ mice, high levels of CCL3 mRNA and protein were detected in the heart tissue during the acute and chronic infection. Survival was not affected by CCL3 deficiency. In comparison with *ccl*3^+/+^, chronically infected *ccl*3^−/−^ mice presented reduced cardiac parasitism and inflammation due to CD8^+^ cells and macrophages. Leukocytosis was decreased in infected *ccl*3^−/−^ mice, paralleling the accumulation of CD8^+^ T cells devoid of activated CCR5^+^ LFA-1^+^ cells in the spleen. Further, *T. cruzi*-infected *ccl*3^−/−^mice presented reduced frequency of interferon-gamma (IFNγ)^+^ cells and numbers of parasite-specific IFNγ-producing cells, while the *T. cruzi* antigen-specific cytotoxic activity was increased. Stimulation of CCL3-deficient macrophages with IFNγ improved parasite control, in a milieu with reduced nitric oxide (NO_x_) and tumor necrosis factor (TNF), but similar interleukin-10 (IL-10), concentrations. In comparison with chronically *T. cruzi*-infected *ccl*3^+/+^ counterparts, *ccl*3^−/−^ mice did not show enlarged heart, loss of left ventricular ejection fraction, QTc prolongation and elevated CK-MB activity. Compared with *ccl*3^+/+^, infected *ccl*3^−/−^ mice showed reduced concentrations of TNF, while IL-10 levels were not affected, in the heart milieu. In spleen of *ccl*3^+/+^ NI controls, most of the CD8^+^ T-cells expressing the CCL3 receptors CCR1 or CCR5 were IL-10^+^, while in infected mice these cells were mainly TNF^+^. Lastly, selective blockage of CCR1/CCR5 (Met-RANTES therapy) in chronically infected *ccl*3^+/+^ mice reversed pivotal electrical abnormalities (bradycardia, prolonged PR, and QTc interval), in correlation with reduced TNF and, mainly, CCL3 levels in the heart tissue. Therefore, in the chronic *T. cruzi* infection CCL3 takes part in parasite persistence and contributes to form a CD8^+^ T-cell and macrophage-enriched cardiac inflammation. Further, increased levels of CCL3 create a scenario with abundant IFNγ and TNF, associated with cardiomyocyte injury, heart dysfunction and QTc prolongation, biomarkers of severity of Chagas' heart disease.

## Introduction

Chagas disease, caused by the protozoan parasite *Trypanosoma cruzi*, affects seven to eight million people in Latin America (1). Most of the infected individuals do not show clinical symptoms during the acute and chronic phases of the disease, however decades later ~30% of them develop a chronic cardiomyopathy, characterized by a mononuclear inflammation enriched in CD8^+^ T-cells and macrophages, fibrosis, electrical abnormalities and heart dysfunction (2–4). The biological mechanisms that govern this differential disease outcome persist unknown. Although inflammatory cells infiltrating the heart may contribute to control parasite growth, they have also been involved in the perpetuation of Chagas' heart disease (5, 6). Further, pro-inflammatory cytokines, interferon gamma (IFNγ) and tumor necrosis factor (TNF) were detected in the heart tissue of chronically infected patients and proposed to be involved in parasite control, but also in tissue damage (3, 7, 8). Moreover, the intensity of a systemic inflammatory profile with high serum levels of TNF, IFNγ and nitric oxide (NO_x_) has been associated with the severity of Chagas' heart disease (9). Treatment of *T. cruzi*-infected macrophages and cardiomyocytes with TNF and IFNγ induced increased the production of the CC-chemokines CCL3/MIP-1α (macrophage inflammatory protein-1α), CCL4/MIP-1β and CCL5/RANTES (regulated upon activation, normal T cell expressed and secreted), which play a pivotal role in parasite growth control (10–12). However, CC-chemokines acting on their receptors CCR1/CCR5 have been involved in myocarditis formation and cardiomyocyte lesion in acute and chronic *T. cruzi* infection (13–16). Nevertheless, the role of the CC-chemokine family members in cardiac tissue inflammation and injury was not clarified.

CCL3 is proposed as a requirement for virus-induced inflammatory response, as CCL3-deficient mice were resistant to Coxsackievirus-induced myocarditis (17). CD8^+^ cells were placed as the main source of CCL3, which plays a crucial role in clearance of intracellular pathogens (18). Therefore, CCL3 became a molecule of interest to be explored in the pathophysiology of the *T. cruzi*-elicited CD8-enriched myocarditis. Although CCL3 serum levels were increased in few patients with severe disease Chagas' heart disease, there was no significant difference in comparison with non-infected individuals (19). Treatment of acutely infected mice with neutralizing anti-CCL3 antibodies led to increased trend in the number of amastigote nests and revealed that this CC-chemokine is involved in macrophage recruitment to cardiac tissue (20). Pre-treatment of human and murine macrophages with CCL3 enhanced parasite uptake and killing, involving NO_x_ production (10–12). Further, CCL3 has been proposed to play a role in the pathophysiology and cardiac remodeling of *T. cruzi* infection, as the dyskinesis of the heart apical region observed in infected wild-type (*ccl3*^+/+^) mice is hampered in infected mice lacking CCL3 (21). CCR1/CCR5, promiscuous receptors for CCL3, CCL4, and CCL5, take part in heart colonization by inflammatory CD8^+^ cells in the acute phase of *T. cruzi* infection (13) and in CCC, controlling fibronectin deposition and parasite load (15). Recently, CCR1^+^ CD14^+^ macrophages were shown to be mainly IL-10^+^, while CCR5^+^ cells were mostly TNF^+^. Further, CCR1^+^ cells were linked to protection, while CCR5^+^ cells were associated with heart tissue injury in experimental CCC (16). However, the participation of the CC-chemokines in CCC has not yet been unveiled. Therefore, using *ccl3*^+/+^ and *ccl*3^−/−^ mice, *in vivo* and *in vitro* approaches and blockage of the CC-chemokine receptors CCR1/CCR5 (Met-RANTES therapy), we explored the role of CCL3 in *T. cruzi* growth control, establishment of the cytokine profile in the heart tissue and CCC pathophysiology, analyzing biomarkers of cardiomyocyte injury, heart dysfunction and electrical abnormalities.

## Materials and Methods

### Ethics Statement

This study was carried out in strict accordance with the recommendations of the Guide for the Care and Use of Laboratory Animals of the Brazilian National Council of Animal Experimentation (http://www.sbcal.org.br/) and Federal Law 11.794 (October 8, 2008). The Institutional Committee for Animal Ethics of Fiocruz (CEUA-Fiocruz-L004/09; LW-10/14) approved all experimental procedures used in the present study. All presented data were obtained from two or three independent experiments.

### Animals

C57BL/6 (H-2^d^) and CCL3-deficient (B6.129P2-Ccl3^tm1Unc^/J) mice were originally purchased from Jackson Laboratories (Sacramento, CA, USA) and matched and maintained in the animal facilities of the Oswaldo Cruz Foundation (CECAL/ICTB/Fiocruz, Rio de Janeiro, Brazil). All mice were maintained under specific pathogen-free conditions with drinking water and chow food *ad libitum*. The wild-type condition (C57BL/6) and CCL3-deficiency were confirmed by genetic characterization of tail samples and using the primers and protocols described at The Jackson Laboratories home page (https://www.jax.org/), thereafter the mice were referred as *ccl*3^+/+^ and *ccl*3^−/−^, respectively.

### Experimental Infection and Met-RANTES Therapy

In all sets of experiments, three to five sex- and age-matched non-infected (NI) controls were analyzed in parallel with 5–10 infected mice according to the experimental protocol. Five- to seven-week-old female mice were infected with the Colombian *T. cruzi* DTU I strain (22) by intraperitoneal injection of 100 blood trypomastigotes, obtained from passage mice to mice every 35 days post-infection (dpi). Parasitemia was estimated in 5 μL of tail vein blood. After the peak of parasitemia, detection of rare circulating trypomastigotes marked the onset of the chronic phase of infection, as previously described (6). Mortality was weekly registered.

Groups of three to five mice were subcutaneously inoculated daily with 0.1 mL of *in vivo* injection-grade saline (BioManguinhos, Rio de Janeiro, RJ, Brazil) or saline containing 10 μg of Met-RANTES, a CCR1/CCR5 partial antagonist (23), kindly provided by Dr. Amanda Proudfoot (Serono Pharmaceuticals, Geneva, Switzerland), for 30 consecutive days (from 120 to 150 dpi) and analyzed at 150 dpi.

### Reagents and Antibodies

For immunohistochemistry (IHC), the polyclonal antibody recognizing *T. cruzi* antigens was produced in our laboratory (LBI/IOC-Fiocruz, Brazil). Purified anti-F4/80 antigen (clone F4/80) antibody was purchased from CALTAG Laboratories (Burlingame, CA). Supernatants were home made with anti-mouse CD8a (53-6.7) and anti-mouse CD4 (GK1.5) hybridomas. Anti-CCL3 polyclonal antibody produced in rabbit was a kind gift of Dr Mauro Teixeira (Universidade Federal de Minas Gerais, Brazil). In our IHC studies, we also used the monoclonal antibodies anti-IFNγ (R4-6A2, BD PharMingen, USA) and anti-perforin (CB5.4, Alexis Biochemicals, San Diego, CA, USA), a polyclonal anti- inducible nitric oxide synthase (iNOS/NOS2) antibody (Cayman Chemical, USA), anti-rat immunoglobulin from DAKO (Glostrup, Denmark), and the biotinylated anti-rabbit immunoglobulin and peroxidase-streptavidin complex from Amersham (England). Appropriate controls were prepared by replacing the primary antibodies with purified rat immunoglobulin or rabbit normal serum (Sigma, USA). For *in vivo* cytotoxicity assays we used a cell trace ^TM^CFSE cell proliferation kit (C34554) for flow cytometry (Invitrogen, Carlsbad, CA, USA). For flow cytometry studies using mouse cells, FITC- or PECy7-conjugated anti-TCR (clone H57.597) were purchased from Southern Biotech (Birmingham, AL, USA). PE-conjugated anti-TCR (clone H57.597), FITC- and APC-conjugated anti-mouse CD8a (53-6.7), PE-cy7-conjugated anti-LFA-1 (CD11a/CD18b, clone 2D7), FITC-conjugated anti-LFA-1 (CD11a/CD18b, clone M17/4), PECy-7-conjugated anti-TNF (MP6-XT22), FITC-conjugated anti-Pfn (clone 11B11), PECy-7-conjugated anti-IFNγ (XMG1.2) and PE-conjugated anti-CCR5 (clone C34-3448) were purchased from BD PharMingen (San Diego, CA, USA). Anti-CCR1-PerCp (clone sc-6125) was obtained from Santa Cruz Biotechnology (Dallas, TX, USA). APC-conjugated anti-IL-10 (clone LRM9104) was obtained from CALTAG (Burlingame, CA, USA). Appropriate controls were prepared by replacing the primary antibodies with their respective isotypes, which were also obtained from BD PharMingen (San Diego, CA, USA) or from Southern Biotech (Birmingham, AL, USA). All the antibodies (final concentrations: 1.5–20 μg/mL) and reagents were used according to the manufacturers' instructions.

### RT-PCR Assay for Detection of Chemokine mRNA

RNA was isolated from heart of mice by acid guanidinium thiocyanate-phenol-chloroform extraction: RNA STAT-60^TM^. Reverse transcriptase-polymerase chain reaction conditions, primer sequences used for detection of CCL3/MIP-1α, and PCR product size have been published elsewhere (24). The PCR product and molecular weight marker were electrophoresed in 6% polyacrylamide gel and stained with silver nitrate. Densitometry of gels was carried out on a Densitometer CS-9301PC (Shimadzu, Japan). The PCRs were standardized using hypoxanthine-guanine phosphoribosyl transferase (HPRT). Data are shown as relative CCL3 mRNA expression.

### Chemotaxis Assay

Leukocyte migration was measured as previously described (25), using a 24-well chemotaxis chamber (6.5 mm of diameter and 3-μm of pore-Falcon-BD, USA). Lower wells were loaded with 0.6 mL (1000 ng/mL) of the recombinant mouse CC-chemokines (CCL3 #450MA, CCL4/MIP-1β #451MB and CCL5/RANTES #478MR, R&D Systems, USA) prepared in RPMI 0.5% BSA. The polyethylene terephthalate membrane filter (#3492, B&D System, USA) pre-coated with 50 μL of purified fibronectin (10 μg/mL, #12173–019, GibcoBRL, USA) was placed between the upper and lower wells. Suspensions of peripheral blood mononuclear cells were prepared by pooling 1.5 mL heparinized individual samples (three animals per group) and performing Ficoll–Hypaque (*d* = 1.077 g/mL) separation, as previously described (24). A suspension containing 10^6^ cells/100 μL was added to the upper wells and the chemotaxis chamber was incubated at 37°C in humidified air with 5% CO_2_ for 3 h. After the incubation period, the lower chamber migrating cells were collected, centrifuged, resuspended in 50μL and counted. The results are expressed as percentage of migrating cells compared with migrating cells in the absence of chemokines as controls.

### Immunohistochemistry (IHC)

Groups of five infected and three age-matched control mice were sacrificed under anesthesia at 28 and 120 dpi. The hearts and spleens of the mice were removed, embedded in tissue-freezing medium (Tissue-Tek, Miles Laboratories, Elkhart, IN, USA), and stored in liquid nitrogen for analysis by immunohistochemistry. Serial 3-μm thick cryostat sections were fixed in cold acetone and subjected to indirect immunoperoxidase staining, as previously described (6, 26). For visualization of the reaction we used the Dako liquid DAB plus substrate chromogen System - PT (K346811-2, Dako, Carpinteria, CA, USA). The positively stained areas for *T. cruzi* antigens^+^ areas and the numbers of positive CD4^+^, CD8^+^, F4/80^+^, IFNγ^+^, and perforin (Pfn)^+^ cells in 100 microscopic fields (50 mm^2^) were counted in heart sections. Tissue sections were stained and numbers of iNOS/NOS2^+^ cells in 100 microscopic fields (50 mm^2^) were counted in heart sections, as previously shown (26). The stained area was analyzed for CCL3 expression and cell types (CD4^+^, CD8^+^, F4/80^+^) in 25 microscopic fields (12.5 mm^2^) in two sections per heart tissue and spleens were evaluated with a digital morphometric apparatus. The images were digitized using a Sight DS-U3 color-view digital camera adapted to an Eclipse Ci-S microscope and analyzed with NIS Elements BR version 4.3 software (Nikon Co., Tokyo, Japan). In a set of experiments, the free software ImageJ (NIH, USA) was used to analyze the percentage of stained area for CCL3 and cell types, as well as the percentage of overlay for IHC-stained areas. According to the analyzed parameters, the data are presented as numbers of positive areas (parasite nests), cells per 100 microscopic fields or percentage of stained area.

### Preparation of Cardiac Tissue Homogenate

Heart tissue was immersed in PBS solution (50 mg/0.5mL) containing complete protease inhibitor cocktail and Nonidet-P40 1% (Sigma, St. Louis, MO, USA). Tissue extract was obtained after homogenization using tissue grinder (T10BS1-5G, Ultra Turrax IKA, Germany) speed 8,000 rpm for 2 min, on ice. The homogenate was centrifuged at 14,000 × g 4°C for 10 min and the supernatant was aliquoted and frozen until use for quantification of cytokines.

### Cytokine Determination by ELISA

The concentrations of cytokines in the cardiac tissue were evaluated by enzyme-linked immunosorbent assay (ELISA) DuoSet kits (R&D Systems, Minneapolis, MN, USA) for mouse IFNγ, TNF, IL-10, and CCL3 were used according to manufacturer's instructions. Diluted (1:2 and 1:10 in PBS) tissue extracts were analyzed in duplicates. Standards were 1/2 log dilutions of the recombinant cytokines from 1 pg/mL to 100 ng/mL.

### Cell Preparation and Flow Cytometry Analysis

The mice were sacrificed by blood draining under anesthesia, the harvested spleens were minced, and red blood cells were removed using lysis buffer (Sigma-Aldrich, St. Louis, MO, USA). For *ex vivo* analysis, splenocytes were incubated with 5 mg/mL brefeldin A (Sigma, St. Louis, MO, USA) for 4 h at 37°C. The cells were collected, washed, resuspended in PBS containing 2% fetal calf serum and labeled as previously described (6). In all experiments, one color labeled samples were prepared for establishment of the compensation values. The controls for specific labeling were prepared using isotype-matched antibodies. For analysis, 300,000 events per sample were acquired using the Beckman Coulter CyAn 7-Color flow cytometer (Fullerton, CA, USA) or 13-Color CytoFLEX-S (Beckman-Coulter, Houston, TX, USA). After gating in singlets and exclusion of dead cells, the mononuclear cell populations were analyzed using Summit v.4.3 Build 2445 software (Dako, Carpinteria, CA, USA) or CytExpert 2.3 software for Windows (Beckman-Coulter, Indianapolis, IN, USA), as described elsewhere (6). The fluorescence gates were cut in accordance with the labeling controls, respecting the curve inflecions. To identify CD8^+^ T cells bearing CCR5 and LFA-1, the gating strategy was as follows: singlets (R1), dead-cell exclusion (FSC-A × SSC-Lin, R2), TCR × CD8 dot plot (gating on TCR^+^ CD8^+^ cells, R3), LFA1 × CCR5 dot plot were analyzed. To characterize the CD8^+^ T cells expressing IFNγ and Pfn, the gating strategy was: singlets (R1), dead-cell exclusion (FSC-A × SSC-Lin, R2), TCR × CD8 dot plot (gating on TCR^+^ CD8^+^ cells), IFNγ × Pfn dot plot (% in quadrants of single-positive or double-positive cells), as previously shown (6). To identify CD8^+^ T cells expressing CC-chemokine receptors and cytokines, the gating strategy was as follows: singlets (R1), dead-cell exclusion (FSC-A × SSC-Lin, R2), TCR × CD8 dot plot (gating on TCR^+^ CD8^+^ cells), CCR1 × CCR5 (gating on single- and double positive cells), and TNF × IL-10 dot plot (% in quadrants of single- or double-positive cells), as described (16).

### IFNγ Enzyme-Linked ImmunoSpot (ELISpot) Assay

The ELISpot assay for the enumeration of IFNγ-producing cells was performed as previously described (27). The assays were performed in triplicate. The plates were coated (50 μL/mL) with anti-mouse IFNγ (clone R4-6A2; BD PharMingen, San Diego, CA, USA) antibody diluted in PBS (5 μg/mL). Further, total splenocytes used as antigen presenting cells (APC) were primed with H-2K^b^-restricted VNHRFTLV peptide from ASP2 (10 μg/mL) for 30 min at 37°C. After three washings, 3 × 10^5^ primed APC in 100 μL were seed per well and incubated with freshly isolated splenocytes seeded at a suspension of 5 × 10^5^ cells/100 μL per well and were incubated for 20 h at 37°C and 5% CO_2_. Concanavalin A (ConA, 5 μg/mL) was used as a mitogenic stimulant. After three-time washings with warmed PBS and three-times with 0.1% Tween-20 PBS. A biotin-conjugated anti-mouse IFNγ antibody (clone XMG1.2; BD PharMingen, San Diego, CA, USA) was used to detect the captured cytokines. The spots were revealed by respective incubation of the samples with a solution of alkaline phosphatase labeled streptavidin (BD PharMingen, San Diego, CA, USA) and a solution of nitro blue tetrazolium (NBT; Sigma, St. Louis, MO, USA) and 5-Bromo-4-chloro-3-indolyl phosphate (BCIP; Sigma, St. Louis, MO, USA) in Tris buffer (0.9% NaCl, 1% MgCl2, 1.2% Tris in H_2_O). The mean number of spots in triplicate wells was determined for each experimental condition using a CTL OHImmunoSpot A3 Analyzer (Cleveland, OH, USA), and the number of specific IFNγ-secreting T-cells was calculated by estimating the stimulated spot count/10^6^ cells. Representative pictures of the assay have previously been published (6).

### In vivo Cytotoxicity Assay

For the *in vivo* cytotoxicity assays, splenocytes collected from *naive* C57BL/6 mice were treated with red blood cells lysis buffer (Sigma, St. Louis, MO, USA). The cells were washed and divided into two populations that were labeled with the fluorogenic dye carboxyfluorescein diacetate succinimidyl diester (CFSE; Molecular Probes, Eugene, OR, USA) at a final concentration of 5 μM (CFSE^high^) or 0.5 μM (CFSE^low^). CFSE^high^ cells were coated with 2.5 μM of the VNHRFTLV ASP2 peptide for 40 min at 37°C. CFSE^low^ cells remained uncoated. Subsequently, CFSE^high^ cells were washed and mixed with equal numbers of CFSE^low^ cells before intravenous injection (1–2 × 10^7^ cells per mouse) into *T. cruzi*-infected C57BL/6 recipients that were sedated with diazepam (20 mg/Kg). Spleen cells from the recipient mice were collected at 20 h after adoptive cell transfer, red blood cells lysed, cells were washed in PBS and fixed with 1.0% paraformaldehyde. For analysis, 30,000 events were acquired with CyAn-ADP flow cytometer (Beckman-Coulter, Houston, TX, USA) and analyzed using the Summit v.4.3 Build 2445 program (Dako, Glostrup, Denmark). Representative pictures of the assay are shown, and the percentage of specific lysis was determined using the following formula:

$$\begin{array}{l} \frac{\left[\%{\text{CFSE}}^{\text{high}}\text{infected}/\%{\text{CFSE}}^{\text{low}}\text{infected}\right]}{\left[\%{\text{CFSE}}^{\text{high}}\text{non}-\text{infected}/\%{\text{CFSE}}^{\text{low}}\text{ non}-\text{infected}\right]}\times \;100\% \end{array}$$

### Macrophage Primary Culture

Peritoneal cells were harvested in serum-free cold DMEM (LCG, Rio de Janeiro, Brazil) from non-infected *ccl3*^+/+^ and *ccl3*^−/−^ mice and washed in serum-free medium at 4°C. Macrophages were resuspended in supplemented DMEM supplemented with fungizone (1 μg/mL), penicillin (1 μg/mL), streptomycin (1 μg/mL) and 10% heat-inactivated fetal calf serum (FBS; Gibco, USA) at 1 × 10^6^/mL, and 0.2 mL were dispensed onto 13 mm glass coverslips placed in a well of a 24-well plate (Falcon, EUA). Cells were allowed to adhere for 2 h at 37°C in the presence of 95% humidity and 5% CO_2_, and then washed three-times with serum-free warmed DMEM, and 1.0 mL of supplemented DMEM was added to each well. The plates were incubated 2 h at 37°C in the presence of 95% humidity and 5% CO_2_ and in the absence or presence of 10 ng/mL of murine recombinant IFNγ (eBioscience, USA). The macrophages were washed and then cultured in the absence or presence of live trypomastigotes obtained from Vero cell line as previously described (28), using a 10:1 parasite:macrophage ratio in a 1.0 mL of supplemented DMEM per well. After 4 h, the supernatants were collected and stored for cytokines and NO_x_ evaluation, the coverslips were gently washed in warmed PBS, fixed in methanol and stained using Giemsa (Merck, Germany), dehydrated in acetone/xylol, mounted in Entellan (Merck, Germany) and assessed using a light microscopy (Nikon, USA). The percentage of cells containing intracellular forms of *T. cruzi* were determined using a light microscope evaluating 200 cells per coverslips. In a set of experiments, peritoneal cells were harvested in serum-free cold DMEM from non-infected and chronically (120 dpi) infected *ccl3*^+/+^ and *ccl3*^−/−^ mice and washed in serum-free medium at 4°C. Cells were allowed to adhere (2 × 10^5^/well) in a 24-well plate for 2 h at 37°C in the presence of 95% humidity and 5% CO_2_, and then washed three-times with serum-free warmed DMEM. After addition of 1.0 mL of supplemented DMEM, the cells were cultivated for 24 h. The supernatants were collected and stored for NO_x_ evaluation.

### Nitric Oxide Quantification

Nitric oxide (NO_x_) concentration was assessed by detection of nitrite/nitrate in macrophage culture supernatants using Griess reaction. Nitrate and NO_x_ levels in serum samples were determined by using the Griess reagent and vanadium chloride III (29). The sample concentrations were estimated based on standard curves from 0.8–200 μM of NaNO_2_ and NaNO_3_.

### Electrocardiogram (ECG) Registers

All mice were intraperitoneally tranquilized with diazepam (20 mg/Kg) and the transducers were carefully placed subcutaneously according to chosen preferential derivation (DII). The traces were recorded for 2 min using a digital system Power Lab 2/20 that was connected to a bio-amplifier at 2 mV for 1 s (PanLab Instruments, Barcelona, Spain). Filters were standardized to between 0.1 and 100 Hz and traces were analyzed using the Scope software for Windows V3.6.10 (PanLab Instruments, Barcelona, Spain). We measured the heart rate (beats per minute, bpm), the duration of the P wave and QRS, PR and QT intervals in milliseconds (ms). The relationship between the QT interval and the RR interval in the mouse was assessed in all animals. To obtain physiologically relevant values for the heart rate-corrected QT interval (QTc) in units of time (rather than time to a power that is not equal to 1), the observed RR interval (RR0) was first expressed as a unitless multiple of 100 ms, yielding a normalized RR interval, RR100 = RR0/ 100 ms. Next, the value of the exponent (y) in the relationship QT0 = QTc × RRy100 was assessed, with QT0 indicating the observed QT and the unit for QTc being ms. The natural logarithm was computed for each side of this relationship [(QT0) = In (QTc) + yln (RR100)]. Thus, the slope of the linear relationship between the log-transformed QT and RR100 defined the exponent to which the RR interval ratio should be raised to correct QT for heart rate (6).

### Transthoracic Echocardiography

For analysis of cardiac function through echocardiography mice were trichotomized in precordial region, sedated with 1.5% isoflurane gas in oxygen with flow 1 L/min, and examined with a Vevo 770 (Visual Sonics, Canada) coupled to a 30 MHz transducer. Cardiac geometry was made using two-dimensional mode images acquired for measurement of internal area of heart cavities (right and left ventricles). Long axis in systole and diastole and left ventricular ejection fraction (LVEF) were determined using Simpson's method.

### Creatine-Kinase Detection

The activity of the creatine kinase myocardial band (CK-MB) is a marker of myocardial injury associated with CCC severity in experimental models (29–32). CK-MB activity was measured with commercial kits (cat. 2010075K, Kovalent do Brasil Ltda, São Gonçalo, RJ, Brazil), as previously described (6). The assay was adapted for reading in a microplate spectrophotometer (ASYS Hitech GmbH, Eugendorf, Austria) to allow the study of small quantities of mouse serum according to the manufacturer's recommendations. The optical density at 340 nm was recorded every 2 min for 15 min.

### Statistical Analysis

For statistical analyses, we used the Student's *t-*test to compare two groups. Comparisons between groups were carried out by analysis of variance (ANOVA) followed by the Bonferroni *post hoc* test. The Kaplan-Meier method was employed to compare survival rates of the studied groups. All statistical tests were performed with GraphPad Prism 8.0.1 (La Jolla, CA, USA). Data are expressed as the arithmetic mean ± SE. Differences were considered statistically significant when *p* < 0.05.

## Results

### Increased CCL3 Levels Are Detected in the Cardiac Tissue of Acute and Chronically T. cruzi-Infected Mice

**Figure 1:**
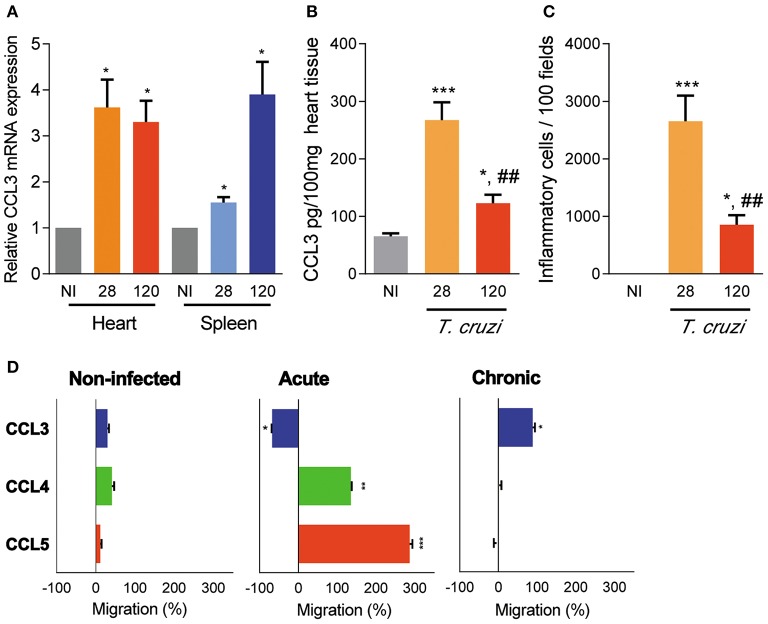
CCL3 expression in the heart and spleen tissues and CC-chemokine-induced chemotaxis of circulating cells of *T. cruzi*-infected C57BL/6 mice. Mice were infected with 100 trypomastigote forms of the Colombian *T. cruzi* strain and analyzed during the acute (28 dpi) and chronic (120 dpi) phases. **(A)** The hearts were collected; mRNA expression was determined by RT-PCR with primers specific for the CCL3 and standardized against HPRT, at 28 and 120 dpi. Data are shown as relative CCL3 mRNA expression. **(B)** Extracts of heart tissues were prepared and CCL3 concentrations estimated by ELISA. Data are expressed as ng of CCL3 per 100 mg of heart tissue. **(C)** Hearts were collected and analyzed by IHC for CD4^+^, CD8^+^, and F4/80^+^ cells. The numbers of total inflammatory cells in 100 microscopic fields are shown. **(D)** *Ex vivo* migration of peripheral blood mononuclear cells in the presence of the CC-chemokines CCL3, CCL4 and CCL5, at 28 and 120 dpi. The migration assay was performed using three mice per group. Data are shown as percentage of migration (%) induced by CC-chemokines compared to migration in absence of stimulus. The assay was performed in triplicate for each point and for each mouse sample. Each experimental group consisted of three mice non-infected (NI) mice and five to seven *T. cruzi*-infected *ccl3*^+/+^ mice in two independent experiments. The data are represented as means ± SE. *p < 0.05, **p < 0.01, and ***p < 0.001, comparing *T. cruzi*-infected and NI mice; ^##^*p* < 0.01, comparing *T. cruzi*-infected mice at 28 and 120 dpi (ANOVA Bonferroni posttest).

In comparison with non-infected (NI) mice, increased expression of CCL3 mRNA transcripts was detected in the heart tissue of acutely infected C57BL/6 *ccl3*^+/+^ mice at 28 dpi and persisted during the chronic phase of infection (120 dpi). Similarly, CCL3 mRNA expression was upregulated in the spleen of acute and chronically *T. cruzi*-infected mice (Figure 1A). Further, in the acute phase of infection CCL3 protein levels were increased in the cardiac tissue and, although reduced, CCL3 expression persisted elevated in the chronic infection (Figure 1B). The IHC study in serial sections of spleen tissues showed that in NI controls and chronically (120 dpi) C57BL/6 infected mice the expression of CCL3 protein preferential overlay with CD8^+^ and, mainly, F4/80^+^ cells (Figure S1). In addition, IHC analyses of cardiac tissues of NI controls revealed low expression of CCL3, which is upregulated in the tissues of chronically *T. cruzi*-infected mice, mainly associated with endothelial and cardiac cells (Figure S2A). Further, CD8^+^ and F4/80^+^ (macrophage marker) cells are mainly located in CCL3^+^ areas of the heart tissue (Figures S2A–C). Importantly, the intensity of heart tissue inflammation paralleled the CCL3 levels in the cardiac tissue in acute and chronically *T. cruzi*-infected mice (Figure 1C).

### CCL3 Controls Cell Migration in the Chronic Phase of T. cruzi Infection

The migration potential of circulating cells of acutely (28 dpi) and chronically (120 dpi) *T. cruzi*-infected *ccl3*^+/+^ mice was assessed by *ex vivo* migration assay using a modified Boyden chamber with a filter coated with fibronectin, allowing cell adhesion and transmigration. The peripheral blood mononuclear cells obtained from NI mice barely migrated toward CCL3, CCL4 and CCL5 (Figure 1D). However, circulating mononuclear cells of acutely infected mice migrated in the presence of CCL4 and CCL5 but did not migrate toward CCL3, which retained the cells on the upper chamber (Figure 1D). Conversely, peripheral blood mononuclear cells from chronically infected mice respond to the chemotactic property of CCL3, albeit did not respond to CCL4 and CCL5 (Figure 1D), supporting a role for CCL3 cell retention in a tissue in the acute phase of and in cell migration in the chronic *T. cruzi* infection.

### CCL3 Is Not Essential for T. cruzi Dissemination Control and Establishment of the Chronic Phase of Infection

**Figure 2:**
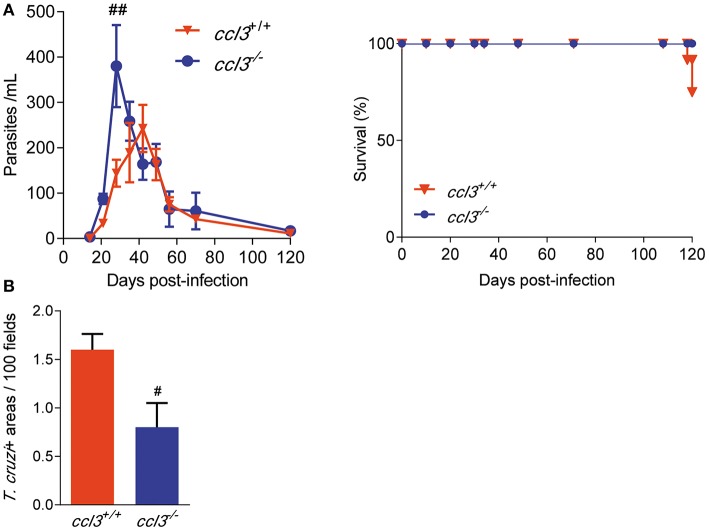
Effects of CCL3 deficiency on parasitemia, survival and heart parasitism in *T. cruzi*-infected mice. *ccl3*^+/+^ and *ccl3*^−/−^ mice were infected with 100 trypomastigote forms of the Colombian *T. cruzi* strain, parasitemia and death were recorded weekly. At 120 dpi, hearts were collected, included in resin and stained for IHC. **(A)** Parasitemia and survival curves of *T. cruzi*-infected *ccl3*^+/+^ and *ccl3*^−/−^ mice. Deaths were registered weekly. **(B)** Heart tissue parasitism was analyzed by IHC and antigen positive areas counted. The data are represented as means ± SE. ^#^*p* < 0.05 and ^##^*p* < 0.01, comparing *T. cruzi*-infected *ccl3*^+/+^ and *ccl3*^−/−^ mice (ANOVA Bonferroni posttest). Data represent two independent experiments with seven to nine infected mice per group (*t*-Student test, ANOVA Bonferroni posttest).

To explore the role of CCL3 in *T. cruzi* parasitemia, survival and control of tissue infection, *ccl3*^+/+^ and *ccl3*^−/−^ mice were infected with 10^2^ parasites of the Colombian *T. cruzi* strain. Although *ccl3*^−/−^ mice showed higher number of circulating parasites in the early acute infection (28 dpi), independently of the CCL3 status all infected mice controlled the parasitemia and achieved the chronic phase of infection (Figure 2A). At 120 dpi, 100% of the *ccl3*^−/−^ and 75–80% of the *ccl3*^+/+^ *T. cruzi*-infected mice survived (Figure 2A). In the acute phase of infection (28 dpi), the *T. cruzi*-infected *ccl3*^−/−^ mice showed an increase in the number of parasite^+^ areas in the cardiac tissue in comparison with infected *ccl3*^+/+^ mice (Figure S3), supporting a role for CCL3 in resistance to acute *T. cruzi* infection. At 120 dpi, when parasite nests are rarely detected in the cardiac tissue of *ccl3*^+/+^ mice, corroborating previous data (6), heart parasitism was lower in *ccl3*^−/−^ infected mice (Figure 2B).

### Reduced Cardiac Inflammation in Chronically *T. cruzi*-Infected CCL3-Deficient Mice

**Figure 3:**
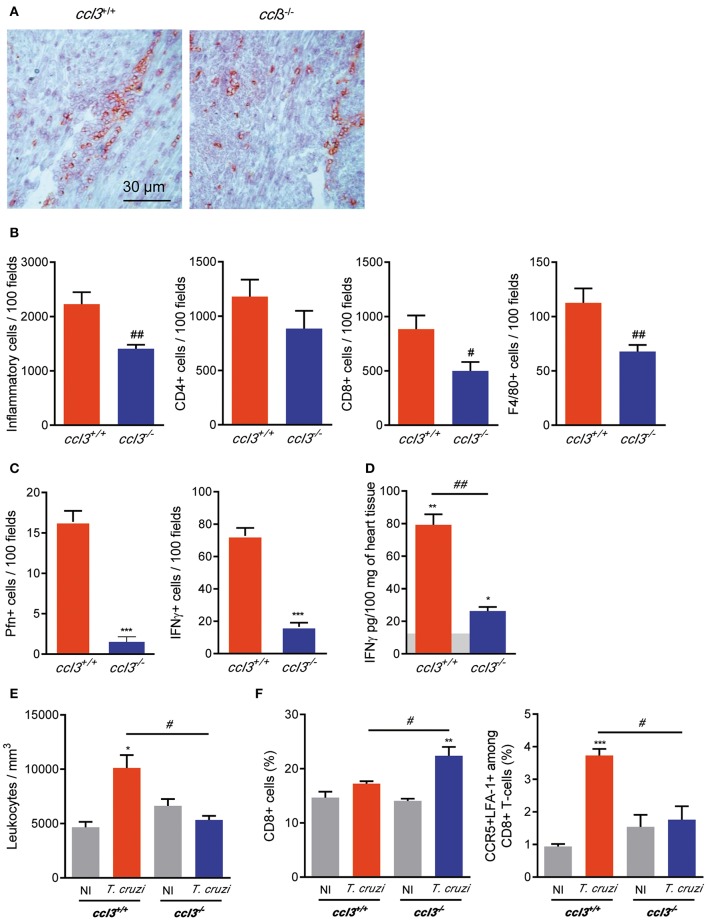
Inflammation and IFNγ concentrations in heart tissue, leukometry and CD8^+^ T-cells in the spleen of chronically *T. cruzi*-infected mice. *ccl3*^+/+^ and *ccl3*^−/−^ mice were infected with 100 trypomastigote forms of the Colombian *T. cruzi* strain and analyzed at 120 dpi. **(A)** Representative sections of heart tissues of infected *ccl3*^+/+^ and *ccl3*^−/−^ mice were IHC for CD8 marker. Horizontal bar indicates 30 μm. **(B)** IHC data showing total inflammation and the presence of CD4^+^, CD8^+^, and F4/80^+^ (macrophages) cells in 100 microscopic field of heart tissue of *T. cruzi*-infected *ccl3*^+/+^ and *ccl3*^−/−^ mice. **(C)** IHC data showing the presence of Pfn^+^ and IFNγ^+^ in 100 microscopic field of heart tissue of *T. cruzi*-infected *ccl3*^+/+^ and *ccl3*^−/−^ mice. **(D)** Hearts of NI and infected mice *ccl3*^+/+^ and *ccl3*^−/−^were collected, extracts were prepared and IFNγ concentrations were estimated by ELISA. Horizontal gray bar shows cytokine levels in non-infected age-matched control mice (means). Data are expressed as ng of CCL3 per 100 mg of heart tissue. **(E)** Peripheral blood was collected; red blood cells were lysed with Turk's reagent and the total leukocytes counted. **(F)** Flow cytometric analysis reveals the percentage of total CD8^+^ T-cells and CCR5^+^ LFA-1^+^ among CD8^+^ T-cells in the spleen of *T. cruzi*-infected *ccl3*^+/+^ and *ccl3*^−/−^ mice and respective NI controls. After selection of singlets (FSC-Lin × FSC-Area, R1), dead-cell exclusion (FSC-A × SSC-Lin, R2), TCR × CD8 dot plot (gating on TCR^+^ CD8^+^ cells, R3), LFA1 × CCR5 dot plot were analyzed. Each experimental group consisted of three non-infected mice and four to five *T. cruzi*-infected mice. Experiments were repeated twice. Data are presented as means ± SE. *p < 0.05, **p < 0.01 and ***p < 0.001, comparing *T. cruzi*-infected and NI mice; ^#^*p* < 0.05 and ^##^*p* < 0.01, comparing *T. cruzi*-infected *ccl3*^+/+^ and *ccl3*^−/−^ mice. (*t*-Student test, ANOVA Bonferroni posttest).

To explore the participation of CCL3 in CCC, we assessed the intensity and cell composition of the *T. cruzi*-elicited myocarditis. At 120 dpi, the *T. cruzi*-infected *ccl3*^−/−^ mice showed reduced heart inflammation composed of CD8^+^ cells compared with infected *ccl3*^+/+^ mice (Figure 3A). Indeed, infected *ccl3*^−/−^ mice presented reduced numbers of cells infiltrating the heart tissue. Although the numbers of CD4^+^ cells infiltrating the heart tissue remained similar, lower numbers of F4/80^+^ and, mainly, CD8^+^ cells were found in the heart tissue of *T. cruzi*-infected *ccl3*^−/−^ mice, in comparison with infected *ccl3*^+/+^ mice (Figure 3B). In comparison with chronically *T. cruzi*-infected *ccl3*^+/+^ mice, *ccl3*^−/−^ mice showed reduced numbers of Pfn^+^ and IFNγ^+^ cells infiltrating the heart tissue (Figure 3C). Further, compared with heart extracts of NI controls, IFNγ concentrations were increased in tissues of infected *ccl3*^+/+^ and *ccl3*^−/−^ mice (Figure 3D). Nevertheless, IFNγ levels were lower in heart tissues of infected *ccl3*^−/−^ mice compared with *ccl3*^+/+^ mice (Figure 3D).

### Reduced Leukocytosis and Increased Retention of CD8^+^ T-Cells in the Spleen of CCL3-Deficient Mice

At 120 dpi, compared with NI control mice *T. cruzi*-infected *ccl3*^+/+^ mice showed an augment in the number of circulating leukocytes. This leukocytosis was not detected in *T. cruzi*-infected *ccl3*^−/−^ mice (Figure 3E), raising the idea that these mice may present impairment in cell activation/migration process. Splenomegaly, characterized by increase in the relative spleen weight and cellularity, is detected in chronically *T. cruzi*-infected mice (6). Here, splenomegaly was similarly detected in *ccl3*^+/+^ (3.29 ± 0.31 mg/g in NI vs. 9.68 ± 0.58 mg/g in infected *ccl3*^+/+^ mice; *p* < 0.001) and *ccl3*^−/−^ (3.78 ± 0.17 mg/g in NI vs. 8.93 ± 0.49 mg/g in infected *ccl3*^−/−^ mice; *p* < 0.001) *T. cruzi*-infected mice. The frequency of CD8^+^ T-cells was alike in NI and chronically *T. cruzi*-infected *ccl3*^+/+^ mice (Figure 3F). However, in comparison with NI (*ccl3*^+/+^ and *ccl3*^−/−^) and infected *ccl3*^+/+^ mice, the frequency of CD8^+^ T-cells was increased in infected *ccl3*^−/−^ mice (Figure 3F), which may indicate retention of CD8^+^ T-cells in the spleen of chronically infected CCL3-deficient mice. In contrast with the high frequency of CCR5^+^ LFA-1^+^ among the splenic CD8^+^ T-cells (R1/R2/R3 gated) detected in *T. cruzi*-infected *ccl3*^+/+^ mice, a remarkable decrease in the frequency of the CCR5^+^ LFA-1^+^ CD8^+^ T-cell population was detected in infected *ccl3*^−/−^ mice (Figure 3F). These data indicate that the reduced numbers of CD8^+^ cells in the heart tissue and circulating leukocytes may reflect the retention of CD8^+^ T-cells in the spleen associated with reduction in the frequency of the potentially able to migrate CCR5^+^ LFA-1^+^ CD8^+^ T-cells in *T. cruzi*-infected *ccl3*^−/−^ mice.

### Inflammatory and Cytotoxic Functions of *T. cruzi*-Specific CD8^+^ T-Cells Are Influenced by CCL3

**Figure 4:**
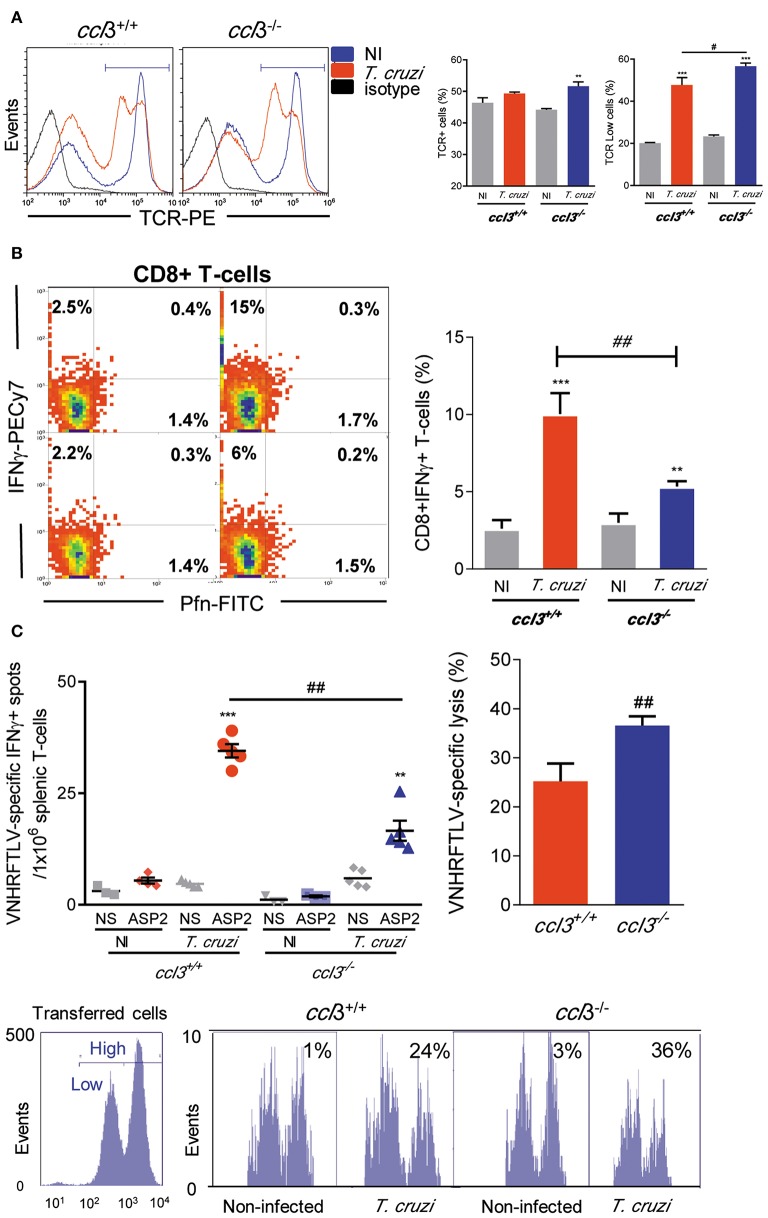
Cell phenotype and effector inflammatory/cytotoxic functions of splenic CD8^+^ T-cells. *ccl3*^+/+^ and *ccl3*^−/−^ mice were infected with 100 trypomastigote forms of the Colombian *T. cruzi* strain and analyzed at 120 dpi. **(A)** TCR expression in splenocytes. After selection of singlets (FSC-Lin × FSC-Area, R1), dead-cell exclusion (FSC-A × SSC-Lin, R2), TCR histograms (representative figures), graphs show total TCR^+^ cells (%) and TCR^Low^ cells (%). **(B)** Presence of Pfn^+^ and IFNγ^+^ CD8^+^ T-cells. After selection of singlets (FSC-Lin × FSC-Area, R1), dead-cell exclusion (FSC-A × SSC-Lin, R2), TCR × CD8 dot plot (gating on TCR^+^CD8^+^ cells, R3), representative Pfn × IFNγ dot plots are shown. Graph shows CD8^+^ IFNγ^+^ T-cells (%). **(C)** The numbers of CD8^+^ IFNγ^+^ were determined by *ex vivo* ELISpot stimulating splenocytes with the H-2K^b^-resctricted ASP2 VNHRFTLV peptide. Bars represent the *in vivo* specific cytotoxicity lysis detected in *T. cruzi*-infected *ccl3*^+/+^ and *ccl3*^−/−^ mice. Each experimental group consisted of three NI mice and four to five *T. cruzi*-infected mice. Representative histogram profiles of *in vivo* cytotoxicity assay showing the specific lysis of in NI controls and *T. cruzi*-infected *ccl3*^+/+^ mice of H-2K^b^-resctricted VNHRFTLV peptide-labeled CFSE^high^ cells compared with non-stimulated CFSE^low^ cells, at 120 dpi. Experiments were repeated twice. Data are presented as means ± SE. **p < 0.01 and ***p < 0.001, comparing *T. cruzi*-infected and NI mice; ^#^*p* < 0.05 and ^##^*p* < 0.01, comparing *T. cruzi*-infected *ccl3*^+/+^ and *ccl3*^−/−^ mice. (ANOVA Bonferroni posttest, *t*-Student test).

At 120 dpi, there was an increase in the frequency of TCR^+^ cells in the spleen of *T. cruzi*-infected *ccl3*^−/−^ mice compared with NI counterpart controls, what is not detected in *ccl3*^+/+^ infected mice (Figure 4A). However, the intensity of TCR expression was reduced on cells of infected *ccl3*^+/+^ and, mainly, *ccl3*^−/−^ mice (Figure 4A). The frequencies of single-positive Pfn^+^ and double-positive IFNγ^+^ Pfn^+^ were not modified after infection of *ccl3*^+/+^ and, mainly, *ccl3*^−/−^ mice. Although the increase in the frequency of splenic IFNγ^+^ CD8^+^ T-cells has been shown in infected *ccl3*^+/+^ and *ccl3*^−/−^ mice, compared with respective NI controls, this was lower in infected *ccl3*^−/−^ mice (Figure 4B). The recognition of the H-2K^b^-restricted ASP2 peptide VNHRFTLV by specific CD8^+^ T-cells has been linked to protective immune response (6, 32). Thus, we evaluated the potential effector function studying IFNγ-producing using *ex vivo* ELISpot assay and the CD8^+^ T-cell cytotoxic activity *in vivo*. At 120 dpi, reduced numbers of VNHRFTLV-specific IFNγ-secreting CD8^+^ T-cells was detected in the spleen of *T. cruzi*-infected *ccl3*^−/−^ mice, compared with infected *ccl3*^+/+^ mice (Figure 4C). This was a condition associated with the absence of CCL3, as ELISpot response to ConA stimulation of splenic cells also led to diminished numbers of IFNγ-secreting cells detected in both NI and *T. cruzi*-infected *ccl3*^−/−^ mice (Figure S4). Conversely, there was an increase in the specific lysis of target cells by the VNHRFTLV-specific cytotoxic CD8^+^ T-cells in *T. cruzi*-infected *ccl3*^−/−^ mice, compared with the cytotoxic activity in infected *ccl3*^+/+^ mice (Figure 4C). Overall, the lack of CCL3 was associated with reduced IFNγ-secreting and enhanced cytotoxic effector activities by splenic CD8^+^ T-cells in chronic *T. cruzi* infection.

### Macrophages of CCL3-Deficient Mice Are More Susceptible to Infection, but in the Presence of IFNγ Control Parasite in a Less Inflammatory Scenario

**Figure 5:**
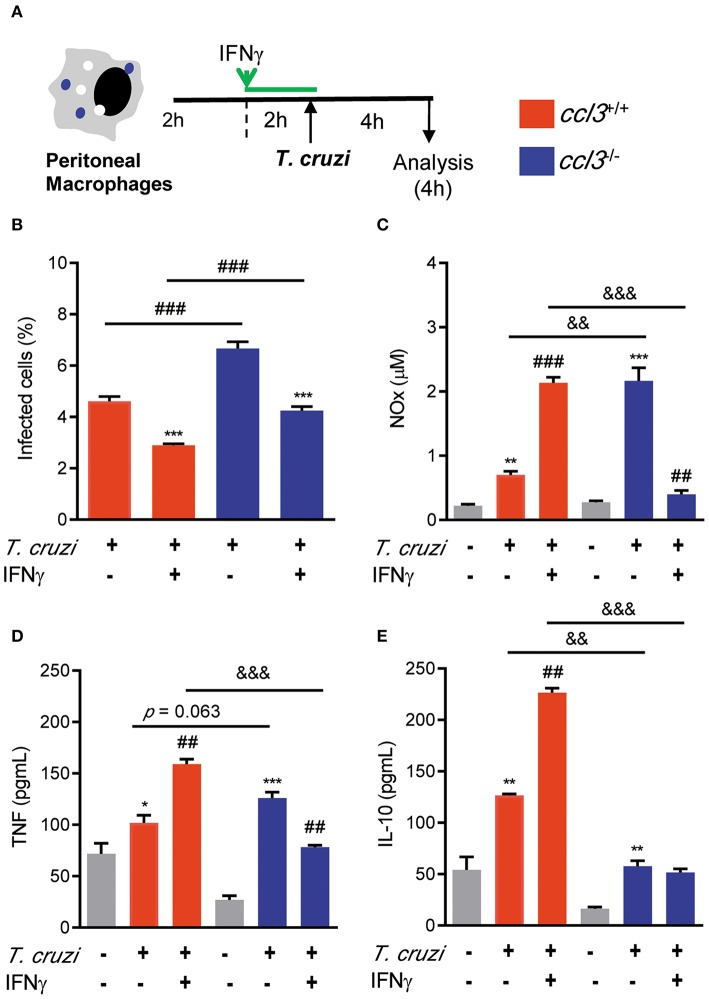
Effects of CCL3 deficiency on macrophages infection ratio and production of NO_x_ and cytokines. **(A)** Peritoneal macrophages from *ccl3*^+/+^ or *ccl3*^−/−^ mice were harvested and allowed to adhere on coverslips (2 h), pre-treated or not treated with IFNγ (2 h) and infected (4 h) by trypomastigote forms of the Colombian strain (MOI of 10:1). **(B)** Percentages of infected cells were quantified. **(C)** Concentrations of NO_x_ in culture supernatants were determined by Griess reagent. Concentrations of **(D)** TNF and **(E)** IL-10 were evaluated using ELISA. Results are representative of three independent experiments performed in triplicates. Data are presented as means ± SE. *p < 0.05 and **p < 0.01, ***p < 0.001, comparing infected with non-infected cells or comparing IFN-treated with not treated. ^##^*p* < 0.01 and ^###^*p* < 0.001, comparing *T. cruzi*-infected *ccl3*^+/+^ and *ccl3*^−/−^ macrophages or infected or non-infected cells. ^&&^*p* < 0.01, ^&&&^*p* < 0.01. (ANOVA Bonferroni posttest, *t*-Student test).

In models of acute infection and in *in vitro* experiments, CCL3 is crucial for control of *T. cruzi* infection, in a NO-dependent manner (11, 12). Here, at 28 dpi there is a similar increase in the numbers of iNOS/NOS2^+^ cells in the heart tissue of *T. cruzi*-infected *ccl3*^+/+^ and *ccl3*^−/−^ mice, compared with NI counterpart controls. At 120 dpi, a similar decay in the numbers of iNOS/NOS2^+^ cells were seen (Figure S5A). Comparable high levels of NO_x_ were detected in the serum of infected *ccl3*^+/+^ and *ccl3*^−/−^ mice, at 120 dpi (Figure S5B). However, peritoneal macrophages obtained from chronically infected *ccl3*^−/−^ mice produced higher levels of NO_x_, compared with macrophages of infected *ccl3*^+/+^ mice (Figure S5C). Therefore, we checked the role of macrophages obtained from CCL3-deficient mice to control *T. cruzi* and to produce the inflammatory mediator NO_x_ and the cytokines TNF and IL-10 (Figure 5A). Peritoneal macrophages obtained of NI *ccl3*^+/+^ mice were susceptible to *in vitro* infection by trypomastigote forms of the Colombian strain (Figure 5B), which triggered the production of NO_x_, TNF and IL-10 (Figures 5C–E). In the presence of IFNγ these macrophages showed reduced frequency of infected macrophages in a milieu with high concentrations of NO_x_, TNF and IL-10 in the supernatants of cultures (Figures 5B–E). Conversely, macrophages isolated from *ccl3*^−/−^ mice were more susceptible to infection by the Colombian strain trypomastigotes (Figure 5B). These *ccl3*^−/−^ macrophages produced similar amounts of NO_x_ and TNF, but lower concentrations of the regulatory cytokine IL-10, compared with macrophages isolated from *ccl3*^+/+^ mice (Figures 5B–E). Notably, when macrophages obtained from *ccl3*^−/−^ mice were stimulated with IFNγ a significant reduction in the frequency of parasitized macrophages (Figure 5B) paralleled the reduction in the concentrations of NO_x_ and TNF (Figures 5C,D). However, the levels of IL-10 were not modified (Figure 5E). Together, these data support that CCL3 is important for macrophage control of parasite infection and formation of an IFNγ-triggered TNF- and NO-enriched inflammatory milieu.

### CCL3 Deficiency Impairs Heart Enlargement and Loss of Left Ventricular Ejection Fraction in Chronic *T. cruzi*Infection

**Figure 6:**
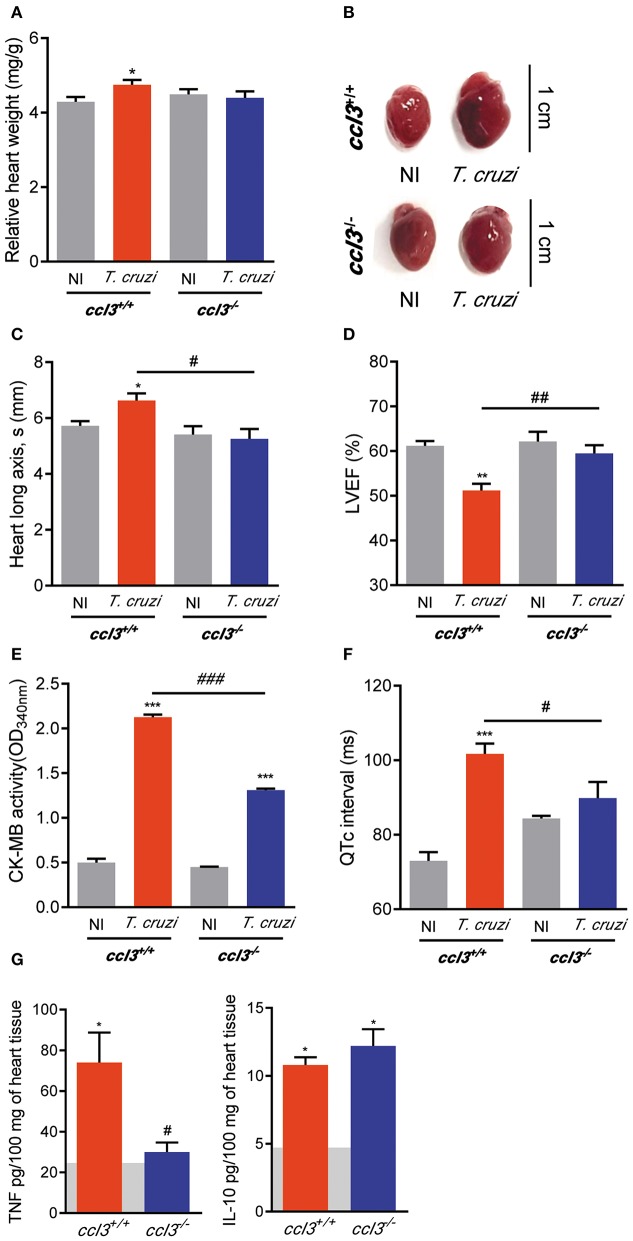
Effects of CCL3 deficiency on CCC main features: heart geometry alterations, loss of LVEF, QTc prolongation and inflammatory/regulatory cytokine unbalance. *ccl3*^+/+^ and *ccl3*^−/−^ mice were infected with 100 trypomastigote forms of the Colombian *T. cruzi* strain and analyzed at 120 dpi. **(A)** Relative heart weight (heart weight in mg/g body) **(B)** Representative pictures of hearts of NI controls and *T. cruzi*-infected *ccl3*^+/+^ and *ccl3*^−/−^ mice. Bars = 1 cm. **(C)** Heart longitudinal axis (mm) in systole (s) was obtained by echocardiography. **(D)** Percentage of left ventricular ejection fraction (LVEF) was determined by echocardiography. **(E)** CK-MB activity in serum determined by biochemical assay. Data represent two independent experiments with three non-infected **(F)** Group data for the ECG records showing QTc intervals (ms). **(G)** Extracts of hearts were prepared and TNF and IL-10 concentrations were estimated by ELISA. Horizontal gray bars show cytokine levels in sex- and age-matched NI control mice (means) and four to five infected mice per group. Data represent two independent experiments. The data are represented as means ± SE. *p < 0.05, **p < 0.01, ***p < 0.001, comparing *T. cruzi*-infected and NI mice; ^#^*p* < 0.05, ^##^*p* < 0.01, ^###^*p*< 0.001, comparing *T. cruzi*-infected *ccl3*^+/+^ and *ccl3*^−/−^ mice. (*t*-Student test, ANOVA Bonferroni posttest).

Next, we analyzed the consequences of CCL3 deficiency for CCC. At 120 dpi, similar body weight was detected in all analyzed mice groups (*ccl3*^+/+^: 21.9 ± 1.3 g in NI vs. 20.6 ± 1.8 g in infected mice, *p* > 0.05; *ccl3*^−/−^: 24.5 ± 3.3 g in NI vs. 21.4 ± 1.6 g in infected *ccl3*^−/−^ mice, *p* > 0.05). Increased relative heart weight was detected in *ccl3*^+/+^ mice compared with NI controls (Figures 6A,B), corroborating previous data (6). However, this heart alteration was not found in *T. cruzi*-infected *ccl3*^−/−^ mice (Figures 6A,B). Further, compared with counterpart NI controls, infected *ccl3*^+/+^ mice showed alterations in heart geometry, revealed as augment in the heart longitudinal axis during systole (Figure 6C). Importantly, this abnormality was not detected in *ccl3*^−/−^ infected mice (Figure 6C). When compared with sex- and age-matched NI counterparts, *ccl3*^+/+^mice chronically infected by the Colombian strain showed decrease in LVEF (Figure 6D). Notably, LVEF values of *T. cruzi*-infected *ccl3*^−/−^ mice resembled their counterpart NI controls (Figure 6D). Altogether, our data support that infected *ccl3*^−/−^ mice were protected of the abnormalities of these geometrical and functional abnormalities detected in infected *ccl3*^+/+^ mice.

### CCL3 Deficiency Reduced Cardiac Tissue Damage and Prevents the Development of QTc Prolongation in Chronic T. cruzi Infection

Inspection of CK-MB isoenzyme activity serum, a marker of cardiomyocyte lesion and cardiomyopathy severity in experimental CCC (30, 32), revealed that CK-MB levels were increased in infected *ccl3*^+/+^ mice compared with NI mice. Importantly, *T. cruzi*-infected *ccl3*^−/−^ mice had lower CK-MB activity levels in serum as compared to infected *ccl3*^+/+^ infected mice (Figure 6E). To explore the participation of CCL3 in *T. cruzi*-elicited cardiac electrical conduction, ECG analyses were carried out in chronically *T. cruzi*-infected *ccl3*^+/+^ and *ccl3*^−/−^ mice. At 120 dpi, bradycardia and increased PR interval were similarly detected in *T. cruzi*-infected *ccl3*^+/+^ and *ccl3*^−/−^ mice, compared with respective NI control groups. In comparison with NI matched controls, the QRS interval remained unaltered in both *ccl3*^+/+^ and *ccl3*^−/−^ mice during the chronic *T. cruzi* infection (Figure S6). Corroborating previous data (16), prolongment of the QTc interval was demonstrated in chronically *T. cruzi*-infected *ccl3*^+/+^ mice. However, the QTc interval was not augmented in *T. cruzi*-infected *ccl3*^−/−^ mice, in comparison with their age- and sex-matched NI controls. Moreover, CCL3 deficiency hampered the augment of the QTc interval in infected *ccl3*^−/−^ mice, compared with infected *ccl3*^+/+^ mice (Figure 6F).

### Selective Reduction of TNF, but Not IL-10, in the Heart Milieu of Chronically Infected CCL3-Deficient Mice

Next, we set out an experiment to analyze the status of the pro-inflammatory TNF and the regulatory IL-10 in heart tissue extracts of *T. cruzi*-infected *ccl3*^+/+^ and *ccl3*^−/−^ mice. In comparison with *ccl3*^+/+^ mice, chronically infected *ccl3*^−/−^ mice showed reduced concentrations of TNF, but similar concentrations of IL-10 in extracts of heart tissue (Figure 6G).

### Augmented TNF Expression Was Mainly Detected in CCR5-Bearing CD8^+^ T-Cells

**Figure 7:**
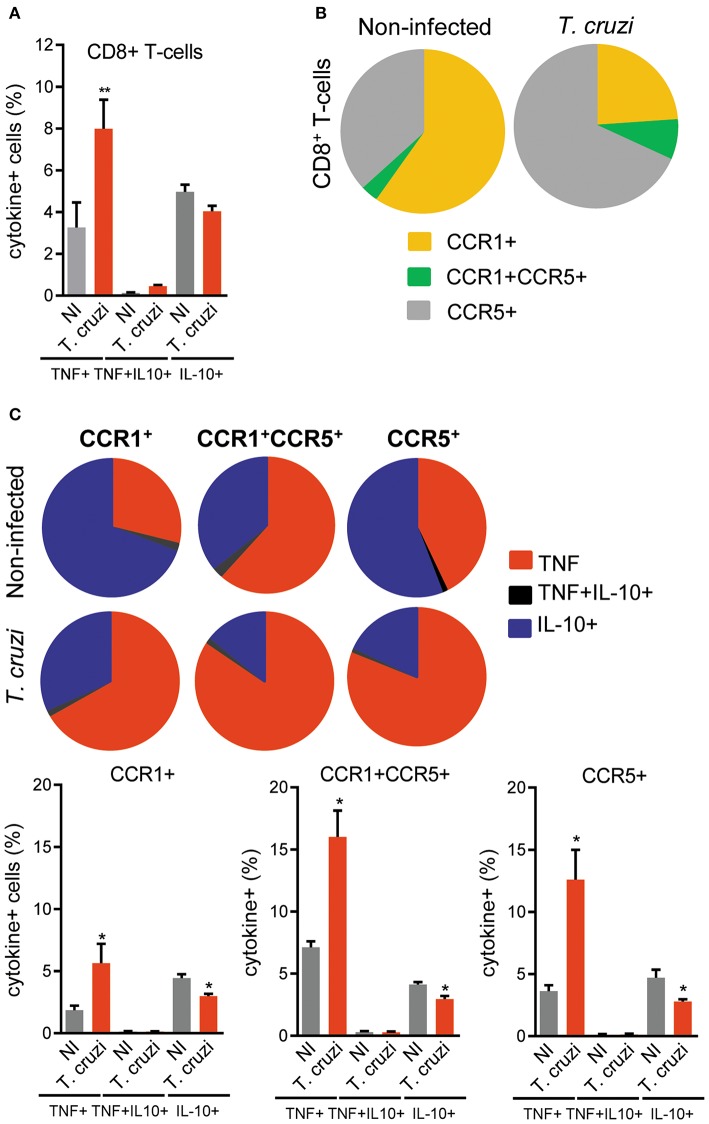
Expression of TNF, IL-10 and CC-chemokine receptors CCR1 and CCR5 by CD8^+^ T-cells in spleen of *T. cruzi*-infected C57BL/6 mice. Mice were infected with 100 trypomastigote forms of the Colombian *T. cruzi* strain and analyzed at 120 days postinfection. Splenocytes were collected and stained for cell surface markers and intracellular cytokines (CD8, CCR1, CCR5; TNF, IL-10). After selection of singlets (FSC-Lin × FSC-Area, R1), dead-cell exclusion (FSC-A × SSC-Lin, R2), TCR × CD8 dot plot (gating on TCR^+^CD8^+^ cells, R3), TNF × IL-10 dot plots were analyzed. **(A)** Graph shows the frequencies of TNF^+^, TNF^+^ IL-10^+^, and IL-10^+^ cells among the CD8^+^ T-cells. **(B)** Pie charts represent the fractions of CD8^+^ T-cells obtained from spleens of NI and *T. cruzi*-infected mice that are CCR1^+^, CCR1^+^ CCR5^+^, and CCR5^+^, as indicated in the legend. **(C)** Pie charts represent the fractions of CCR1^+^, CCR1^+^ CCR5^+^, and CCR5^+^ CD8^+^ T-cells that carry each of the intracellular cytokine phenotypes shown in the legend. Data represent two independent experiments with three NI and four to five infected mice. ^*^P < 0.05, **P < 0.01, NI vs. *T. cruzi* (ANOVA Bonferroni posttest).

Here, we showed that the intensity of the CD8-enriched myocarditis and electrical abnormalities were related to the concentrations of TNF in the cardiac tissue. Thus, we analyzed the cytokine profiles, focused on TNF and IL-10, in CD8^+^ T-cells and, particularly, in CD8^+^ T-cells bearing the CCL3 receptors CCR1 and CCR5 on the cell surface. At 120 dpi, the frequencies of CD8^+^ T-cells were similar in NI controls (12.35 ± 1.3%) and *T. cruzi*-infected (12.35 ± 1.3%) C57BL/6 mice. Compared with NI controls, we observed an increase in the frequency of TNF^+^, but not in IL-10^+^ and TNF^+^ IL-10^+^, among the CD8^+^ T-cells in the spleen of infected mice (Figure 7A). The frequency of CCR1^+^ was reduced (5.9 ± 1.7% in NI vs. 3.7 ± 1.9% infected mice; *p* < 0.05), whereas the frequencies of CCR1^+^ CCR5^+^ (0.34 ± 0.12% in NI vs. 1.2 ± 0.6% infected mice; *p* < 0.001) and CCR5^+^ (3.6 ± 1.2% in NI vs. 10.6 ± 1.5% infected mice; *p* < 0.001) were augmented among the CD8^+^ T-cells (Figure 7B). In NI mice, CCR1^+^ and CCR5^+^ CD8^+^ T-cells showed a predominance of IL-10-expressing cells, while the CCR1^+^ CCR5^+^ were mostly TNF^+^ (Figure 7C). At 120 dpi, we detected a decrease in the frequency of IL-10^+^ cells and an increase in the frequencies of TNF-expressing cells among the CCR1^+^, CCR1^+^ CCR5^+^ and CCR5^+^ CD8^+^ T-cells (Figure 7C). Indeed, TNF/IL-10 ratio tend to be elevated in CCR1^+^ (0.39 ± 0.17% in NI vs. 2.0 ± 1.3% infected mice; *p* = 0.092) and was increased in CCR1^+^ CCR5^+^ (1.6 ± 0.12% in NI vs. 6.8 ± 1.6% infected mice; *p* < 0.01) and CCR5^+^ (0.69 ± 0.16% in NI vs. 4.8 ± 1.9% infected mice; *p* < 0.05) CD8^+^ T-cells. Therefore, *T. cruzi* infection fuels with TNF expression the CD8^+^ T-cells expressing CCR1 and/or CCR5, inflammatory cells potentially able to respond to CC-chemoattractants as CCL3.

### Met-RANTES Therapy Has Beneficial Effects on ECG Abnormalities Associated With Reduction of CCL3 and TNF Levels in the Cardiac Tissue

**Figure 8:**
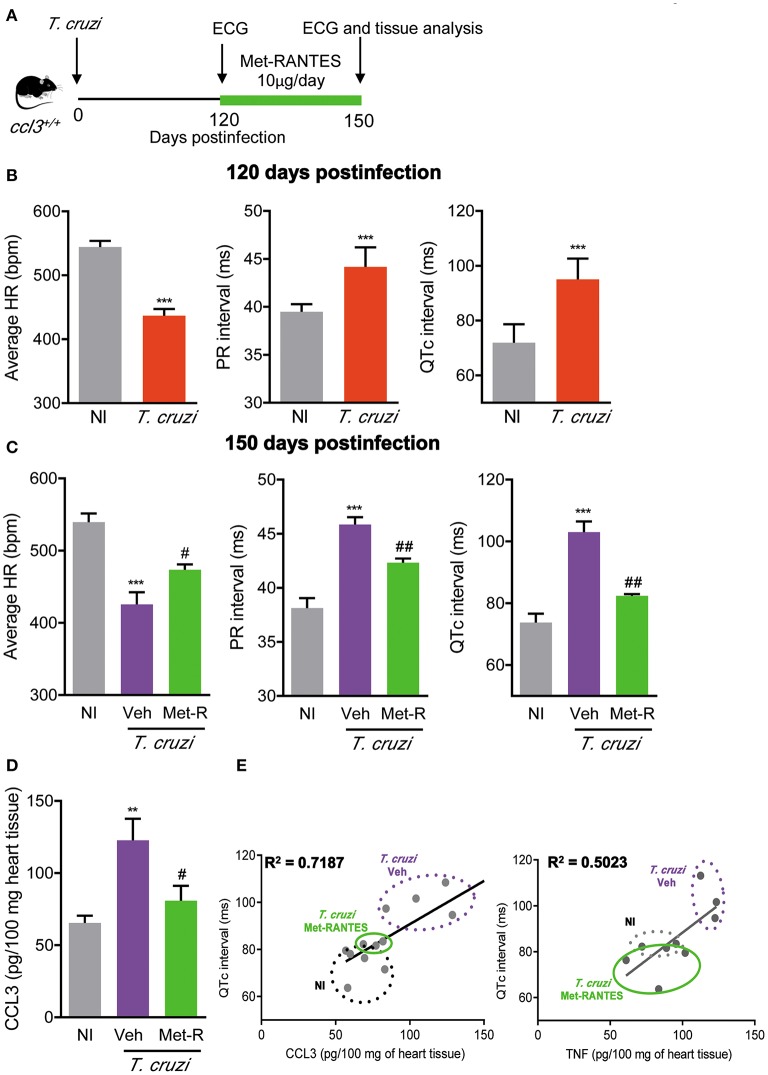
Met-RANTES effects on ECG records and cytokine concentrations in the heart tissue of *Trypanosoma cruzi*-infected mice. **(A)** C57BL/6 mice were infected with 100 trypomastigote forms of the Colombian *T. cruzi* strain and treated from 120–150 dpi with vehicle (Veh) or Met-RANTES (Met-R, 10 μg/mice). At 120 and 150 dpi, ECG registers were recorded. At 150 dpi, hearts were collected for evaluation of CCL3 and TNF concentrations by ELISA. **(B)** Group data for the ECG records showing the average heart rate (beats per minute, bpm), variation in the PR and QTc intervals (ms), at 120 di. **(C)** Group data for the ECG records showing the average heart rate (beats per minute, bpm), variation in the PR and QTc intervals (ms), at 150 dpi. **(D)** CCL3 concentrations in extracts of heart tissue evaluated by ELISA, at 150 dpi. **(E)** Correlations of CCL3 and TNF concentrations in heart tissue extracts with QTc intervals (ms), at 150 dpi. Data represent two independent experiments with five NI and three to four infected mice. The data are represented as means ± SE. **P < 0.01 and ***P < 0.001, *T. cruzi*-infected mice vs. NI; ^#^*P* < 0.05, ^##^*P* < 0.01 Veh-treated vs. Met-R-treated. (*t*-Student test, ANOVA Bonferroni posttest).

In chronic *T. cruzi* infection, macrophages (CD14^+^ CD45R^+^ F4/80^+^ cells) are mostly segregated in CCR5^+^ expressing TNF, while the CCR1^+^ mainly express IL-10 (16). Here, we showed an increased frequency of TNF^+^ cells among CCR1- and CCR5-bearing CD8^+^ T-cells. Therefore, we explored whether the blockage of CCR1/CCR5 with Met-RANTES affects *T. cruzi*-induced ECG abnormalities and the relation with CCL3 and TNF status in the heart tissue. For that, C57BL/6 mice were infected, analyzed at 120 dpi for ECG abnormalities, divided in two groups and treated with vehicle or Met-RANTES. At 150 dpi, animals were analyzed for ECG alterations and cytokine concentrations in the cardiac tissue (Figure 8A). At 120 dpi, average heart rates were reduced and PR and QTc intervals were prolonged, compared with NI controls (Figure 8B). At 150 dpi, in vehicle-treated mice ECG alterations progressed (Figure 8C). Conversely, blockage of CCR1/CCR5 with Met-RANTES more than hampered progression of ECG alterations as partially reversed the bradycardia and the prolonged PR in infected mice. Moreover, Met-RANTES administration almost completely reversed the prolonged QTc intervals present in chronically infected mice (Figure 8C). At 150 dpi, compared with saline-injected mice Met-RANTES therapy reduced chronic myocarditis (774 ± 146 cells/100 fields in saline vs. 547 ± 62 cells/100 fields in Met-R; *p* < 0.01), mostly due to reduction in the numbers of CD8^+^ (338 ± 93 cells/100 fields in saline vs. 228 ± 49 cells/100 fields in Met-R; *p* < 0.05) and F4/80^+^ cells (158 ± 39 cells/100 fields in saline vs. 97 ± 57 cells/100 fields in Met-R; *p* = 0.0744) and less to alteration in the numbers of CD4^+^ cells (278 ± 89 cells/100 fields in saline vs. 221± 39 cells/100 fields in Met-R; *p* > 0.05). Compared with age- and sex-matched NI controls, CCL3 concentrations were increased in the heart tissue of chronically *T. cruzi*-infected vehicle-injected mice, while CCL3 levels were diminished by Met-RANTES therapy (Figure 8D). Importantly, the TNF and, mainly, CCL3 concentrations in the heart tissue were directly correlated with QTc prolongation (Figure 8E).

## Discussion

In the present study, our main data show that in chronic *T. cruzi* infection CCL3 may contribute to parasite persistence and to induce a cardiac inflammation composed mainly of CD8^+^ T-cells and macrophage in a milieu enriched in IFNγ, TNF, and CCL3, associated with cardiomyocyte injury, enlarged heart, LVEF loss and QTc prolongation. Therefore, we bring evidence that CCL3 plays a pivotal role in *T. cruzi*-induced chronic cardiomyopathy.

Initially, we validated our experimental model to study the contribution of CCL3 in the pathophysiology of *T. cruzi-*induced cardiomyopathy. Previous data have demonstrated increased CCL3 expression in the cardiac tissue of C57BL/6 and C3H/He mice infected with the Colombian type I *T. cruzi* strain (24, 33). Corroborating these data, our results disclosed an increase in CCL3 mRNA expression in the hearts and spleens of acute and chronically Colombian-infected C57BL/6 mice. Similarly, increased CCL3 mRNA expression was detected in the heart tissue of C57BL/6 mice and hamsters infected with the Y type II strain (34, 35), rats infected with the CL-Brener type VI strain (36) and dogs infected with the Berenice-78 strain type II strain (37). In addition, we showed high levels of CCL3 protein in the cardiac tissue of acute and chronically infected C57BL6 mice, as previously shown in C3H/He mice (15). Thus, the detection of CCL3 in the heart tissue in experimental models using diverse mammal species and parasite strains supports that it is a conserved feature in *T. cruzi* infection.

Importantly, the intensity of inflammation paralleled the CCL3 concentration in the heart tissue during the acute and chronic phases of *T. cruzi* infection. In an *ex vivo* chemotactic assay, we showed that CCL4 and CCL5 coordinated the migration of circulating cells obtained from acutely infected mice, while CCL3 has selectively driven the migration of blood cells isolated from chronically infected C57BL/6 mice, reinforcing previous findings in C3H/He mice (25). The formation of inflammation in a target tissue is a complex process and other chemokines rather than CCL3 may play a role in chemoattraction, transmigration and positioning of cells in the heart tissue during *T. cruzi* infection (13, 15, 16). Anyhow, our data support that the infection of C57BL/6 mice with the Colombian strain reproduces some aspects involving CCL3 described in other models of Chagas' heart disease, therefore representing a suitable experimental model to approach questions on the participation of CCL3 in the pathophysiology of CCC.

Initially, we challenged the role of CCL3 in parasite control. Compared with the wild-type counterparts, the increased parasitemia and heart parasitism in acutely *T. cruzi*-infected *ccl3*^−/−^ mice supports the premise that CCL3 controls acute infection elicited by diverse pathogens and in different target tissues. Indeed, CCL3 is crucial for control of *Cryptococcus neoformans* infection in lung and brain (38, 39), hepatitis virus infection in the central nervous system (40) and *Listeria monocytogenesis* disseminated infection (41). Importantly, in these cases CCL3 is essential for protective mechanisms involving the recruitment of effector CD8^+^ T-cells and macrophages to the sites of infection, robust early production of cytokines (TNF and IFNγ), induction of cytolytic activity and generation of radical oxygen intermediates. Our work shows that CCL3 deficiency did not impact parasitemia control and survival, as *ccl3*^−/−^ mice survived acute phase when challenged with low inoculum (100 parasites) of the Colombian strain and developed the chronic infection. Thus, for the first time the role of CCL3 controlling growth of an infectious agent is explored in a model of long-term infection (120 dpi). Compared with acutely infected counterparts, the already low-grade *T. cruzi* heart parasitism seen in chronically infected *ccl3*^+/+^ mice is even lower in chronically infected *ccl3*^−/−^ mice. Crucial expression of molecules involved in parasite control are preserved in CCL3-deficient mice as iNOS/NO_2_ expression in the heart tissue and NO_x_ levels in serum. Further, the higher levels of NO_x_ produced by peritoneal macrophages obtained from chronically infected *ccl3*^−/−^ mice may contribute to a more efficient control of parasite systemically, leading to reduced heart parasitism. Thus, our data support that CCL3 may act dualistically, in the acute infection controlling parasite growth and in the chronic phase facilitating, direct or indirectly, heart parasitism. This idea may encounter support in the role played by CCL3 enhancing *T. cruzi* uptake (10), which may favor parasite entrance in host cells and the establishment of infective intracellular forms, in a molecular way that deserves further investigation. Previous findings supported that CCL3 is not essential for *T. cruzi* control in CCL3-vaccinated acutely infected rats (36) or herpes simplex virus control in mice (42). However, our data extend this idea supporting that in the chronic infection CCL3 may favor parasite persistence in the heart tissue, which is associated to tissue inflammation (35), hallmarks of Chagas' heart disease (2–5).

A putative role for CC-chemokines in CCC physiopathology was raised by the demonstration that the concentrations of CCL3 and CCL5 in the cardiac tissue paralleled the intensity of myocarditis in acute and chronically Colombian-infected C3H/He mice (15). CCL3-defficient mice were resistant to Coxsackie virus-induced acute myocarditis seen in infected wild-type mice (17). Here, we showed that although not fully abrogated chronic myocarditis was substantially and selectively reduced (low numbers of CD8^+^ cell and macrophages, unaffected number of CD4^+^ cells) in infected in *ccl3*^−/−^ mice. Importantly, the numbers of Pfn^+^ and IFNγ^+^ cells infiltrating the heart tissue were also reduced in infected *ccl3*^−/−^ mice. These cells are protective for parasite control, but play antagonistic roles in CCC, as Pfn^+^ cells were proposed to be deleterious and IFNγ^+^ protective (6). Therefore, the present data support that CCL3-driven process is crucial for the migration of these distinct cell populations to the heart tissue and establishment of an inflammatory milieu.

The inflammatory CD8^+^ cells found in the heart tissue are mainly blood-born circulating CCR5^+^LFA-1^+^ T-cells, which are activated in secondary lymphoid tissues (6, 15, 24). Reduced number of circulating leukocytes paralleled the accumulation of CD8^+^ T-cells in spleen of chronically infected *ccl3*^−/−^ mice. Moreover, the frequency of splenic CCR5^+^ LFA-1^+^ among CD8^+^ T-cells, a population that gain the blood, enter the heart tissue and play effector functions (6, 15, 43), was reduced in *T. cruzi*-infected *ccl3*^−/−^ mice. These findings support that cell activation, trafficking and colonization of the heart tissue was disturbed in chronically infected CCL3-deficient mice. Interestingly, the upregulation of CCR5 mRNA expression in CD8^+^ T cells is dependent on CCL3 (40). Therefore, our data show that CCL3 is essential for the acquisition of the full migration potential (CCR5 and LFA-1 expression) by CD8^+^ T-cells in *T. cruzi* infection. The presence of CCL3 in the cardiac tissue in *T. cruzi* infection may be considered a molecular signature of the infiltrating inflammatory cells and may drive their activation and effector functions in this tissue. Indeed, CD8^+^ cells, the main source of CCL3 (18), is the major component of the chronic *T. cruzi*-induced myocarditis in humans (2, 3) and experimental models (6, 24, 33). In the spleen, most of the CCL3-reactive areas colocalized with CD8^+^ cells and F4/80^+^ macrophages. In the cardiac tissue, *T. cruzi* infection upregulated CCL3 staining mainly in endothelial and cardiac cells. Indeed, previous work support that *T. cruzi*-infected cardiomyocytes produce CCL3 (34). Further, CD8^+^ and F4/80^+^ cells are localized in areas with intense CCL3 staining in the heart tissue. Therefore, CCL3 may drive full activation/differentiation, as acquisition of CCR5 on cell surface in peripheral tissues and may also coordinate the migration of inflammatory CCR5^+^ CD8^+^ T-cells to the heart tissue in chagasic infection. The colonization of the heart tissue by CCR5^+^ CD8^+^ T-cells (and also CCR5^+^ macrophages) may contribute to create a CCL3-enriched milieu, and critically auto-sustain the infiltration of the heart tissue by these cell, as supported by the partial abrogation of the acute (13) and chronic *T. cruzi* infection-elicited myocarditis (15, 16) by the CCR1/CCR5 antagonist Met-RANTES (23).

CD8^+^ T-cell production of IFNγ and Pfn-mediated cytotoxicity have been linked to protective response in *T. cruzi* infection (6, 32), thus we evaluated the role of CCL3 on these effector functions. TCR profiles were similar in infected *ccl3*^−/−^ mice, although the TCR downregulation, typically detected in chronic *T. cruzi*-infected mice (32), were more evident in *ccl3*^−/−^ mice. The frequencies of Pfn^+^ and Pfn^+^ IFNγ^+^ CD8^+^ T-cells were also similar, but the frequency of IFNγ^+^ was drastically reduced, in chronically infected *ccl3*^+/+^ and *ccl3*^−/−^ mice. Thus, we assessed the impact of CCL3-deficiency on the H-2K^b^-restricted VNHRFTLV-specific effector CD8^+^ T-cells, as the recognition of this ASP2 peptide was associated to protective immunity (6). In the spleen of chronically infected mice, CCL3 deficiency partially impaired CD8^+^ T-cells effector function, reducing IFNγ production after stimulus with the ASP2 *T. cruzi* antigen, in compatibility with the reduced frequency of IFNγ^+^ CD8^+^ T-cells. However, the ASP2-specific cytotoxic activity was increased *ccl3*^−/−^ mice, paralleling the accumulation of CD8^+^ T-cells in spleen, and may contribute to systemic parasite control in the chronic infection, as cytotoxic CD8^+^ T-cells are crucial for parasite control (6, 43).

CCL3 is essential for macrophages recruitment (17). *In vitro*, CCL3 addition to macrophages led to *T. cruzi* infection control, in a NO-dependent manner (11, 12). When infected *in vitro*, macrophages of CCL3 deficient mice showed higher number of intracellular forms of the parasite than macrophages of wild-type mice, as previous data suggested (20). However, our findings support that CCL3 deficiency did not affect the capacity of macrophages to respond to IFNγ-elicited trypanocidal activity. IFNγ plays a key role in resistance to acute *T. cruzi* infection, mainly involving macrophage activation and NO production (44). As expected, in our study IFNγ has driven the activation of wild-type macrophages to release high levels of NO_X_, TNF and IL-10. However, in the absence of CCL3 the addition of IFNγ led to reduction of NO_X_ and TNF levels and did not affect IL-10 production, supporting that CCL3 is a key chemokine controlling the cytokine profile produced by macrophages in response to *T. cruzi* infection.

The severity of Chagas' heart disease is characterized by inflammation enriched in IFNγ- and TNF-expressing cells (7, 8). Interestingly, in the heart tissue of chronically infected *ccl3*^−/−^ mice the numbers of IFNγ^+^ cells were diminished. The concentrations of IFNγ and TNF were also reduced, paralleled by the decreased numbers of CD8^+^ T-cells and macrophages, potential sources of these cytokines. In contrast, the levels of IL-10 were unchanged, regardless of the CCL3 status. Thus, our data indicate that CCL3 is important for the establishment of a TNF- and IFNγ-enriched milieu in the heart tissue during chronic *T. cruzi* infection. In hepatitis virus-infected *ccl3*^−/−^ mice, the IFNγ production by antigen-specific CD8^+^ T-cells isolated from brain and lymph nodes was dramatically reduced (40). Also, previous data support that CCL3 blockade by neutralizing antibodies induced by CCL3-containing plasmid vaccine reduced IFNγ production in heart tissue in acutely infected rats (36). Truly, our data corroborated the idea that CCL3 is pivotal for IFNγ production. Moreover, we extend this finding showing that TNF, but not IL-10, production is impacted by CCL3 status in a way that chronically *T. cruzi*-infected *ccl3*^−/−^ mice have a less inflammatory and, therefore, a more balanced cytokine profile in the cardiac tissue. It is a quite remarkable contribution to understand the physiopathogenesis of the chronic cardiomyopathy triggered by *T. cruzi* infection, as the TNF/IL-10 balance may crucially control the clinical outcome of chronic Chagas disease (7, 16, 45). Altogether, these data support that a new balance of inflammatory/regulatory cytokines, potentially less deleterious, was achieved in the milieu of the cardiac tissue of chronically *T. cruzi*-infected CCL-3 deficient mice.

We have previously shown that chronically Colombian-infected C57BL/6 mice show cardiomegaly, echocardiographic and electrical abnormalities that resembles those found in Chagas' heart disease (6, 26, 32, 46). These features were associated with the intensity of myocarditis and levels of proinflammatory cytokines in the heart tissue (26, 30). Here, we bring evidence that CCL3 status influences heart geometry, as in infected *ccl3*^−/−^ mice enlarged heart and increase in longitudinal axis during systole were not observed. Moreover, in CCL3-deficient mice LVEF was preserved, in contrast with heart dysfunction detected in infected *ccl3*^+/+^ mice. ECG alterations as bradycardia and increased PR interval, indicating a delay in the conduction of electric impulse together with an increase in the duration of ventricular action potential, were similarly detected in chronically *T. cruzi*-infected *ccl3*^+/+^ and *ccl3*^−/−^ mice. Importantly, CCL3 deficiency prevents the augment in QTc interval, a hallmark of chronic *T. cruzi* infection in humans (47, 48) and mice (26). The evaluation of the CK-MB, a biomarker of myocardial injury (49), revealed significant increase in the activity of this isoenzyme in serum of *T. cruzi*-infected C57BL/6 mice in the chronic infection, as previously shown (6, 32). However, these levels were significantly reduced in *T. cruzi*-infected *ccl3*^−/−^ mice in parallel to the reduction in concentrations of inflammatory cytokines in heart tissue and occurred in the presence of a more regulatory milieu, with reduced TNF concentrations and preserved IL-10 levels. Altogether, these data corroborated the idea that a dysregulated inflammatory profile is associated with heart injury and functional and electrical abnormalities (26, 30–32) in chronically infected mice.

In a previous study, we showed that in NI controls and chronically *T. cruzi*-infected mice macrophages (CD14^+^ CD45R^+^ F4/80^+^ cells) are mostly segregated in CCR5^+^ TNF^+^ and CCR1^+^ IL-10^+^ (16). Here, we bring evidence that CCR1^+^ and CCR5^+^ CD8^+^ T-cells are enriched in IL-10-expressing cells in NI controls. Conversely, after *T. cruzi* infection CCR1^+^, CCR1^+^ CCR5^+^ and CCR5^+^ CD8^+^ T-cells are mostly composed of TNF-expressing cells. Therefore, CD8^+^ T-cells expressing CCR1 an CCR5 were not phenotypically segregated as macrophages (16), however *T. cruzi* infection improves the inflammatory TNF-enriched profile. Lastly, considering the role of CCR1/CCR5 in cell migration and action in the heart tissue in experimental *T. cruzi* infection (13, 15), and the beneficial effects of Met-RANTES therapy reducing the TNF-enriched inflammatory milieu in the heart tissue and tissue injury (16), we challenged the effects of Met-RANTES therapy on CCL3 status in the heart and the relation with ECG abnormalities. Importantly, Met-RANTES therapy started after establishment of ECG alterations (at 120 dpi) not only hampered the progression of ECG abnormalities, but also reversed PR and QTc prolongation. Moreover, QTc prolongation was associated with TNF and CCL3 concentrations in the heart tissue of chronically *T. cruzi*-infected mice. The beneficial effect of Met-RANTES on ECG abnormalities and decrease in TNF and CCL3 concentrations in heart paralleled the reduction of myocarditis intensity, mainly due to decrease in the numbers of CD8^+^ and F4/80^+^ cells invading the heart tissue. These data reinforce the potential role of these cells as sources of TNF and CCL3 in CCC. Prolonged QTc has been associated with heart inflammation enriched in TNF, IL-1, and IL-6 in several human cardiac conditions (48). Further, in the acute phase of C57BL/6 infection by the Colombian strain prolonged QTc has been associated with severe and specific miRNA alterations (50), as well as increased TNF and IFNγ gene expression (51). Blockage of TNF by neutralizing antibody (30), disruption of TNF/TNFR1 signaling by pentoxifylline administration (31) and reduction of TNF expression in the heart tissue by benznidazole therapy (26) reduced the prolonged QTc, reinforcing the participation of TNF-signaling in this abnormality. Our study was limited as we did not approach the CCL3-driven molecular mechanisms promoting heart tissue parasitism in chronic *T. cruzi* infection. More importantly, our findings place CCL3 as a key chemokine in chronic *T. cruzi* infection, as a putative sponsor for the CD8^+^ T-cells and macrophage-enriched myocarditis, in a scenario with abundant IFNγ, TNF and CCL3. Thus, CCL3 may contribute directly or indirectly to trigger crucial CCC features as tissue injury and heart electrical and functional abnormalities. Particularly, for the first time, CCL3 is proposed to contribute to heart dysfunction and QTc prolongation, biomarkers of prognosis for CCC severity and mortality risk in Chagas disease (47, 52). Considering that long QT syndrome and/or heart dysfunction are main features of chronic infectious and non-infections (as diabetes) cardiopathies (48), the importance of our findings transcends the comprehension of the pathophysiology of Chagas disease. Therefore, strategies that reduce CCL3 expression in the heart tissue may improve the prognosis of chronic heart diseases associated with TNF-enriched inflammation, as CCC and long QT syndromes.

## Data Availability Statement

All datasets generated for this study are included in the article/Supplementary Material.

## Ethics Statement

This study was carried out in strict accordance with the recommendations in the Guide for the Care and Use of Laboratory Animals of the Brazilian National Council of Animal Experimentation (http://www.cobea.org.br/) and the Federal Law 11.794 (October 8, 2008). The Institutional Committee for Animal Ethics of Fiocruz (CEUA-Fiocruz LW-10/14, CEUA-IOC L-006/2018) approved all experimental procedures used in the present study. All presented data were obtained from seven independent experiments, due to reduced number of CCL3 mice offered by the animal facilities.

## Author Contributions

JL-V, AS, and DG: conceived and designed the experiments. JL-V, AS, DG, GV-P, IP, LB, IR, and HM: performed the experiments. AS, IP, DG, GV-P, IR, and JL-V: analyzed the data. DG and JL-V: wrote the manuscript. RG and JL-V: acquired mice and reagents. All authors read and revised the manuscript.

### Conflict of Interest

The authors declare that the research was conducted in the absence of any commercial or financial relationships that could be construed as a potential conflict of interest.
