# Supplementary figures and images for: CCL3/Macrophage Inflammatory Protein-1α Is Dually Involved in Parasite Persistence and Induction of a TNF- and IFNγ-Enriched Inflammatory Milieu in Trypanosoma cruzi-Induced Chronic Cardiomyopathy
Source: Front Immunol. 2020 Mar 3;11:306. doi: 10.3389/fimmu.2020.00306 (PMC7063958; doi:10.3389/fimmu.2020.00306)

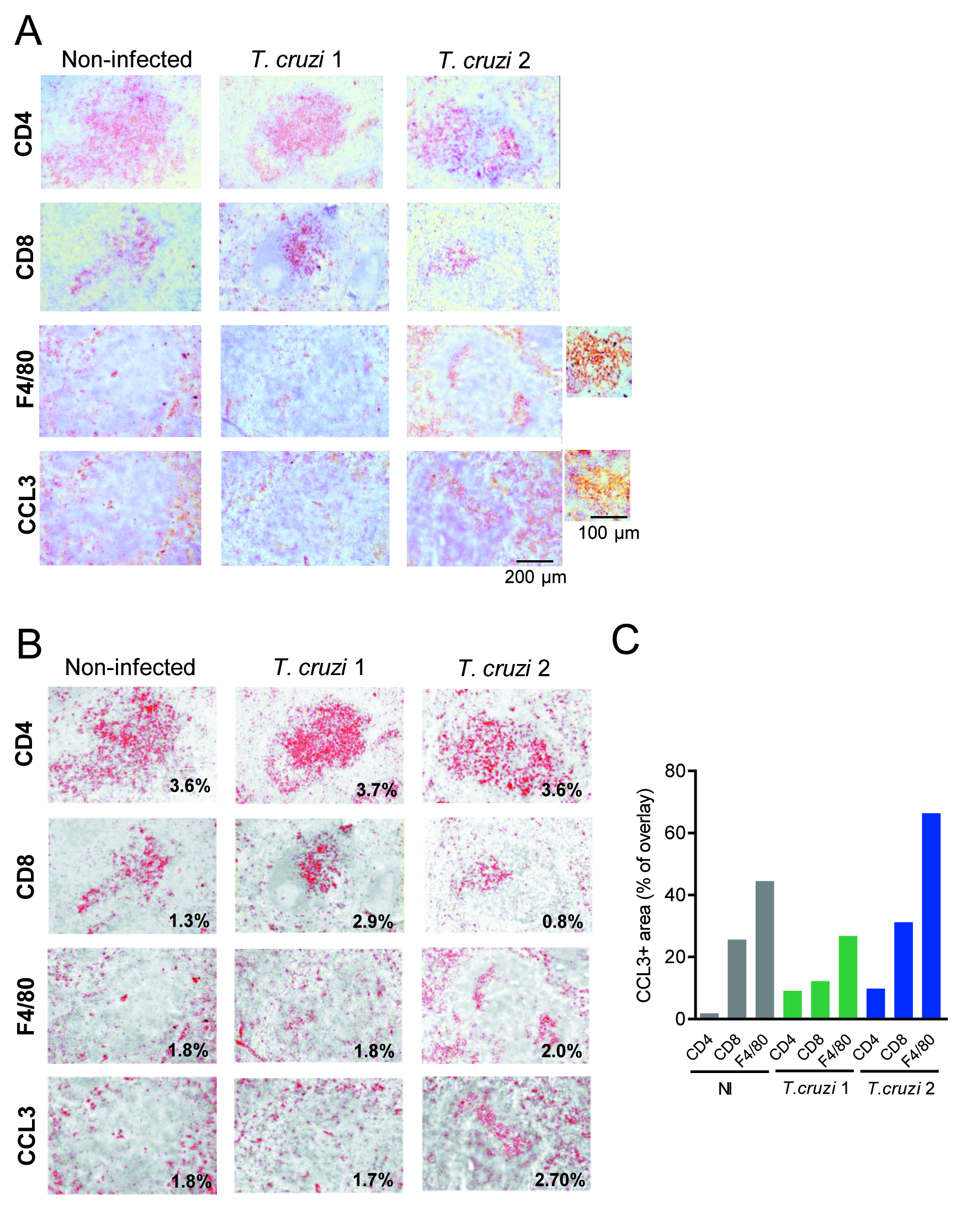

Supplement: Supplementary file 1 [file Image_1.TIF]

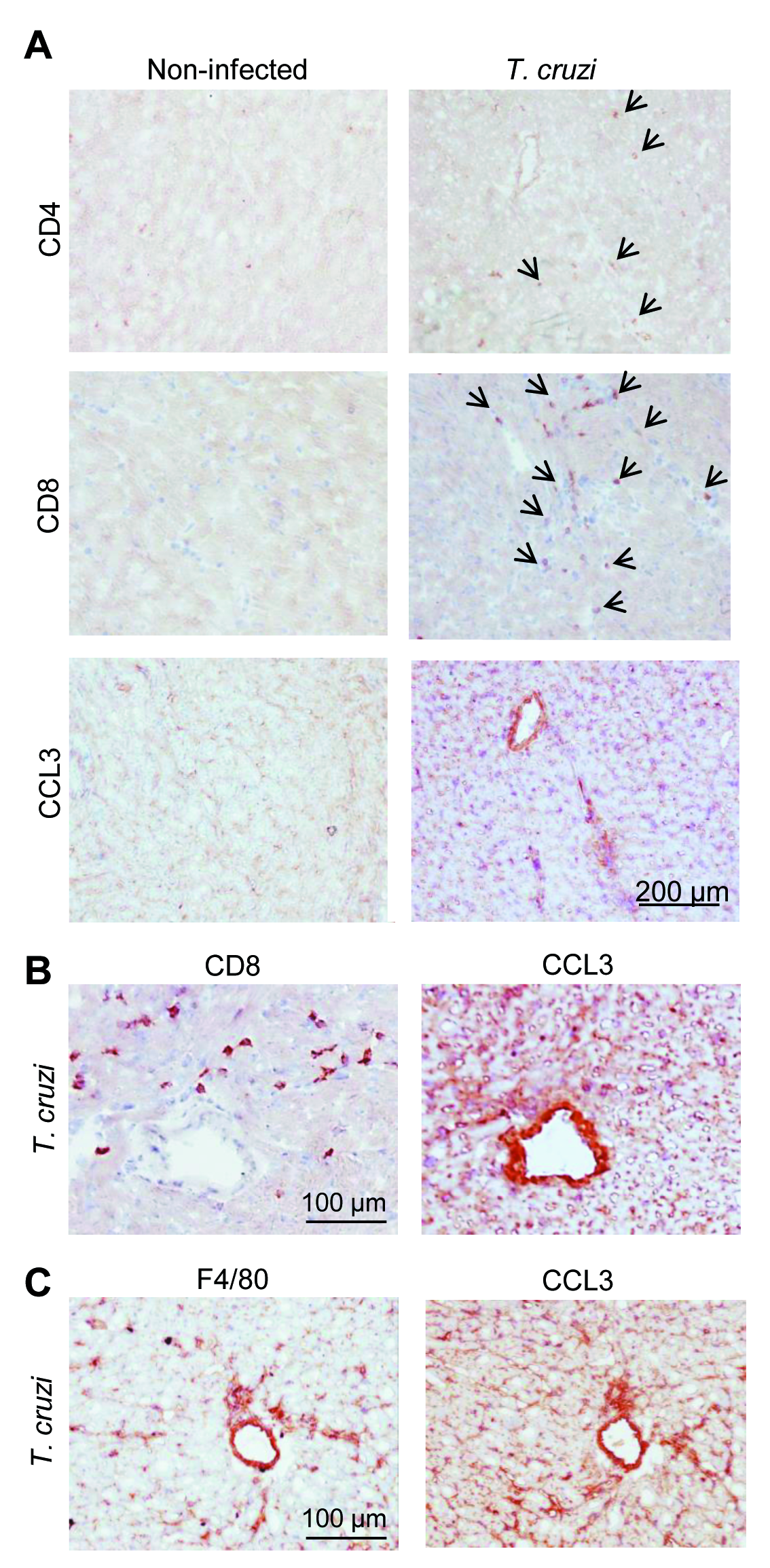

Supplement: Supplementary file 2 [file Image_2.TIF]

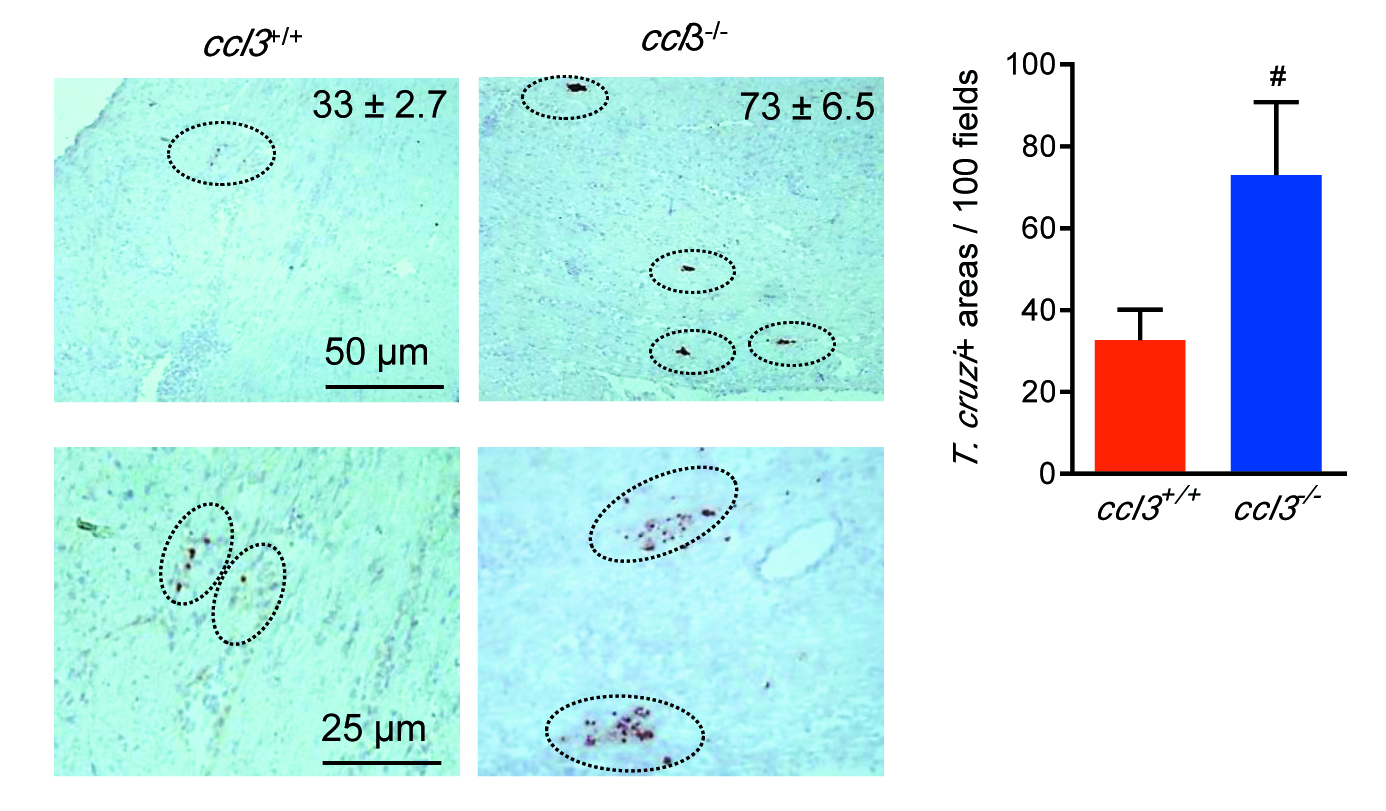

Supplement: Supplementary file 3 [file Image_3.TIF]

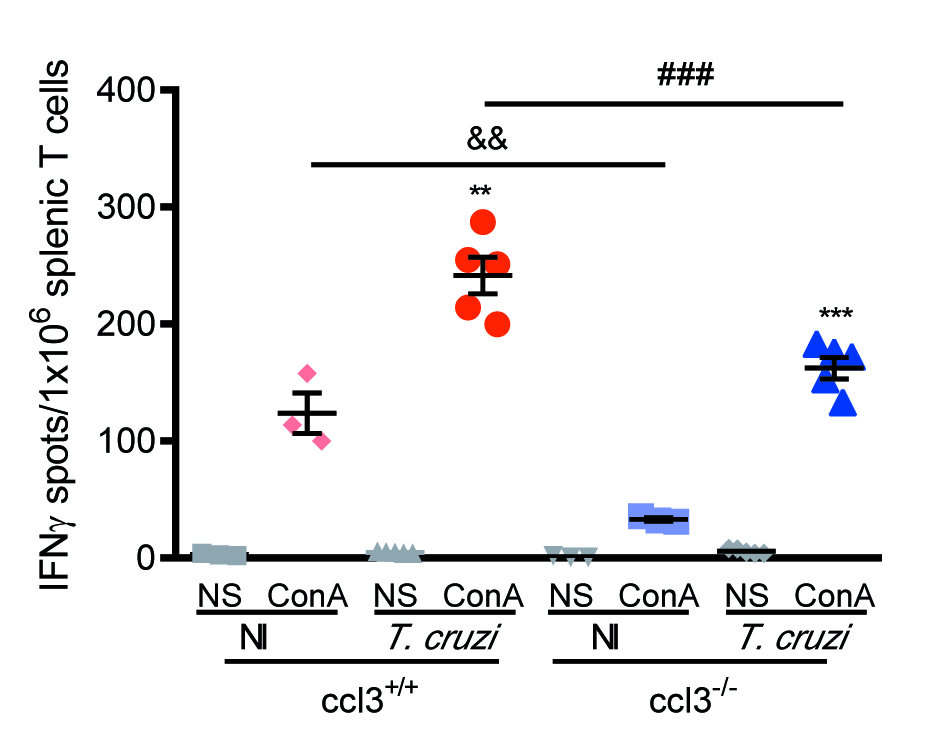

Supplement: Supplementary file 4 [file Image_4.TIF]

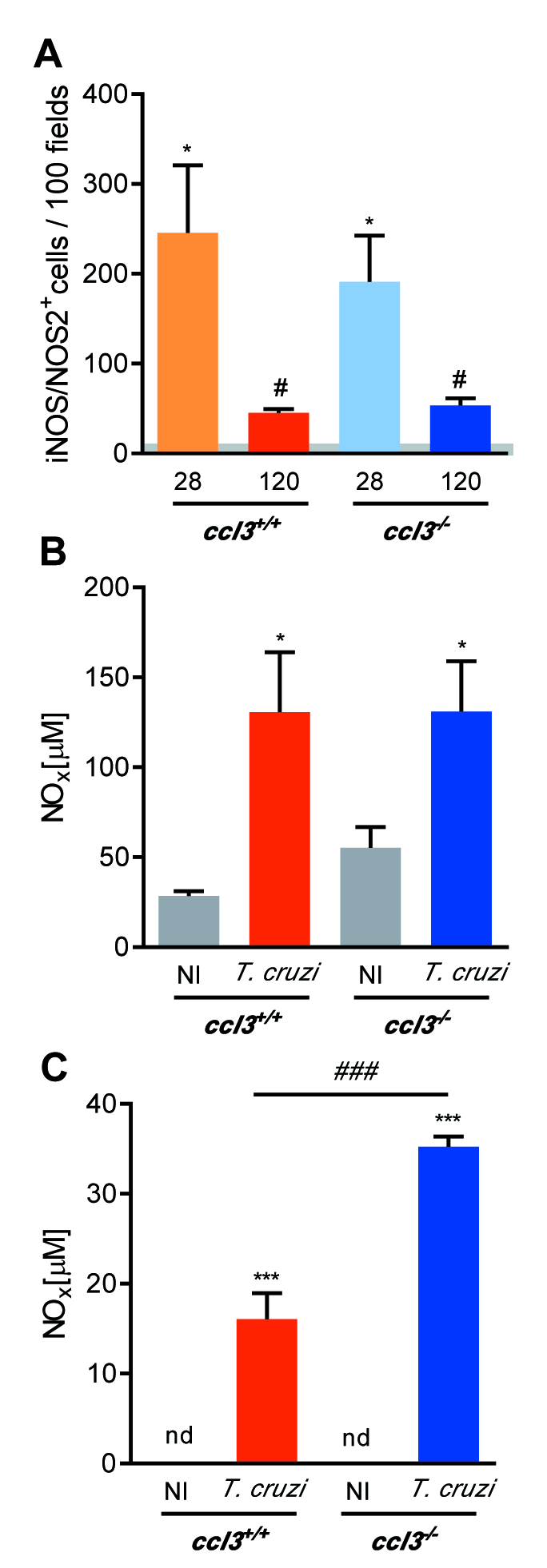

Supplement: Supplementary file 5 [file Image_5.TIF]

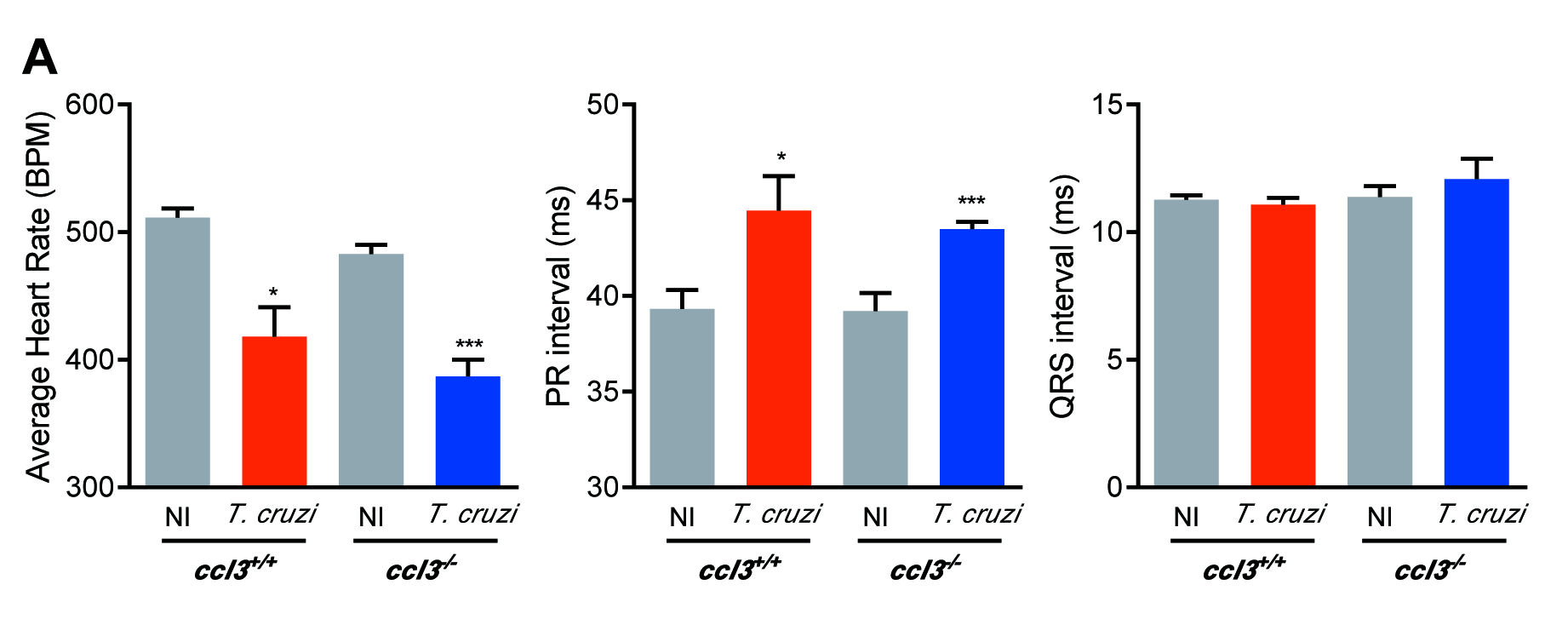

Supplement: Supplementary file 6 [file Image_6.TIF]
