# Supplementary material for: CCL3/Macrophage Inflammatory Protein-1α Is Dually Involved in Parasite Persistence and Induction of a TNF- and IFNγ-Enriched Inflammatory Milieu in Trypanosoma cruzi-Induced Chronic Cardiomyopathy
Source: Front Immunol. 2020 Mar 3;11:306. doi: 10.3389/fimmu.2020.00306 (PMC7063958; doi:10.3389/fimmu.2020.00306)
Supplement: Supplementary file 7 [file Table_1.DOCX]

**SUPPLEMENTARY MATERIAL LEGENDS**

**Figure S1.** Expression of CCL3 and cell types in spleen of NI and *T. cruzi*-infected *ccl3*^+/+^ mice. Mice were infected with 100 trypomastigote forms of the Colombian *T. cruzi* strain and analyzed at 120 dpi. (**A**) Representative pictures of serial sections of spleens of NI and infected *ccl3*^+/+^ mice submitted to IHS for CD4^+^, CD8^+^ and F4/80^+^ (macrophages) cells and CCL3. Horizontal bars indicate 100µm or 200µm. (**B**) Representative pictures of serial sections of spleens analyzed using ImageJ software. Percentages of stained areas are shown. (**C**) Graph shows percentages of overlay of IHS staining for CCL3 and CD4^+^, CD8^+^or F4/80^+^cells in representative mice.

**Figure S2.** Expression of CCL3 and cell types in heart tissue section of NI and *T. cruzi*-infected *ccl3*^+/+^ and *ccl3*^-/-^ mice. Mice were infected with 100 trypomastigote forms of the Colombian *T. cruzi* strain and analyzed at 120 dpi. (**A**) Representative pictures of sections of hearts of NI and infected *ccl3*^+/+^ and *ccl3*^-/-^ mice submitted to IHS for CD4^+^and CD8^+^ cells and CCL3. Horizontal bar indicates 200µm. Arrow heads indicate positive cells. (**B**) Representative pictures of serial sections of hearts submitted to IHS for CD8^+^ cells and CCL3. Horizontal bar indicates 100µm. (**C**) Representative pictures of serial sections of hearts submitted to IHS for F4/80^+^cells and CCL3. Horizontal bar indicates 100µm.

**Figure S3.** Effects of CCL3 deficiency on heart parasitism in *T. cruzi*-infected *ccl3*^+/+^ and *ccl3*^-/-^ mice. Mice were infected with 100 trypomastigote forms of the Colombian *T. cruzi* strain. At 28 dpi, hearts were collected, included in resin, stained for immunohistochemical assay and antigen positive areas counted. Representative pictures are shown. Horizontal bars represent 50 or 25 µm. The data are represented as means ± SE. ^#^, *p*<0.05. Data represent two independent experiments with 3 infected mice per group. (t-Student test).

**Figure S4.** Effector inflammatory function of splenic CD8^+^ T-cells of chronically *T. cruzi*-infected *ccl3*^+/+^ and *ccl3*^-/-^ mice. Mice were infected with 100 trypomastigote forms of the Colombian *T. cruzi* strain and analyzed at 120 dpi. The number of CD8^+^IFNγ^+^ as determined by *ex vivo* ELISpot stimulating splenocytes with ConA mitogen. Each experimental group consisted of 3 NI and 4-5 *T. cruzi*-infected mice. Experiments were repeated twice. ^**^, *p*<0.01 and ^***^, *p*<0.001, comparing *T. cruzi*-infected and NI mice; ###, *p*<0.001, comparing *T. cruzi*-infected *ccl3*^+/+^ and *ccl3*^-/-^ mice; ^&&^, *p*<0.01, comparing NI *ccl3*^+/+^ and *ccl3*^-/-^ mice. (ANOVA Bonferroni posttest).

**Figure S5.** Expression of iNOS/NOS2 in heart tissue, NO_x_ in serum and NO_x_ in culture supernatants in presence or absence of CCL3. *ccl3*^+/+^ and *ccl3*^-/-^ mice were infected with 100 trypomastigote forms of the Colombian *T. cruzi* strain and analyzed at 120 dpi. (**A**) Heart tissue sections were submitted to IHS for detection of iNOS/NOS2 producing cells. Data are shown as numbers of iNOS/NOS2^+^ cells in 100 microscopic fields. Horizontal grey bar shows the numbers of iNOS/NOS2^+^ cells in NI sex- and age-matched control mice (means). (**B**) Concentrations of NO_x_ serum were determined by Griess reagent and vanadium chloride III, using standard curves from 0.8-200 µM of NaNO_2_ and NaNO_3_. Data are shown as µM of NO_x_. (**C**) Peritoneal macrophages obtained from NI and *T. cruzi* mice were cultured for 24 hours and culture supernatants used to evaluate NO_x_ concentrations by Griess reagent, using standard curves from 0.8-100 µM of NaNO_2_ and NaNO_3_. Data are shown as µM of NO_x_. ^*^, *p*<0.05 and ^***^, *p*<0.001, comparing *T. cruzi*-infected and NI mice. #, *p*<0.05, ###, *p*<0.001, comparing *T. cruzi*-infected *ccl3*^+/+^ and *ccl3*^-/-^ mice; (ANOVA Bonferroni posttest).

**Figure S6.** Effects of CCL3 deficiency on electrical records of chronically *T. cruzi*-infected mice. *ccl3*^+/+^ and *ccl3*^-/-^ mice were infected with 100 trypomastigote forms of the Colombian *T. cruzi* strain and analyzed at 120 dpi. Group data for the ECG records showing the average heart rate (beats per minute, bpm), variation in the PR and QRS intervals (ms). ^*^, *p*<0.05 and ^***^, *p*<0.001, comparing *T. cruzi*-infected and NI mice. (ANOVA Bonferroni posttest).
